# Temporal dynamics and drivers of durable HIV viral load suppression and persistent high‐ and low‐level viraemia during Universal Test and Treat scale‐up in Uganda: a population‐based study

**DOI:** 10.1002/jia2.26200

**Published:** 2024-02-08

**Authors:** Joseph Gregory Rosen, Robert Ssekubugu, Larry W. Chang, Victor Ssempijja, Ronald M. Galiwango, Joseph Ssekasanvu, Anthony Ndyanabo, Alice Kisakye, Gertrude Nakigozi, Katherine B. Rucinski, Eshan U. Patel, Caitlin E. Kennedy, Fred Nalugoda, Godfrey Kigozi, Oliver Ratmann, Lisa J. Nelson, Lisa A. Mills, Donna Kabatesi, Aaron A. R. Tobian, Thomas C. Quinn, Joseph Kagaayi, Steven J. Reynolds, Mary Kathryn Grabowski

**Affiliations:** ^1^ Department of International Health Johns Hopkins Bloomberg School of Public Health Baltimore Maryland USA; ^2^ Rakai Health Sciences Program Entebbe Uganda; ^3^ Division of Infectious Diseases Johns Hopkins School of Medicine Baltimore Maryland USA; ^4^ Department of Epidemiology Johns Hopkins Bloomberg School of Public Health Baltimore Maryland USA; ^5^ Clinical Monitoring Research Program Directorate Frederick National Laboratory for Cancer Research Frederick Maryland USA; ^6^ Department of Mathematics Imperial College London UK; ^7^ Division of Global HIV and TB Centers for Disease Control and Prevention Kampala Uganda; ^8^ Department of Pathology Johns Hopkins School of Medicine Baltimore Maryland USA; ^9^ Division of Intramural Research National Institute of Allergy and Infectious Diseases National Institutes of Health Bethesda Maryland USA

**Keywords:** antiretroviral therapy, HIV treatment, HIV viraemia, prospective cohort, sub‐Saharan Africa, Treat All

## Abstract

**Introduction:**

Population‐level data on durable HIV viral load suppression (VLS) following the implementation of Universal Test and Treat (UTT) in Africa are limited. We assessed trends in durable VLS and viraemia among persons living with HIV in 40 Ugandan communities during the UTT scale‐up.

**Methods:**

In 2015–2020, we measured VLS (<200 RNA copies/ml) among participants in the Rakai Community Cohort Study, a longitudinal population‐based HIV surveillance cohort in southern Uganda. Persons with unsuppressed viral loads were characterized as having low‐level (200–999 copies/ml) or high‐level (≥1000 copies/ml) viraemia. Individual virologic outcomes were assessed over two consecutive RCCS survey visits (i.e. visit‐pairs; ∼18‐month visit intervals) and classified as durable VLS (<200 copies/ml at both visits), new/renewed VLS (<200 copies/ml at follow‐up only), viral rebound (<200 copies/ml at initial visit only) or persistent viraemia (≥200 copies/ml at both visits). Population prevalence of each outcome was assessed over calendar time. Community‐level prevalence and individual‐level predictors of persistent high‐level viraemia were also assessed using multivariable Poisson regression with generalized estimating equations.

**Results:**

Overall, 3080 participants contributed 4604 visit‐pairs over three survey rounds. Most visit‐pairs (72.4%) exhibited durable VLS, with few (2.5%) experiencing viral rebound. Among those with any viraemia at the initial visit (23.5%, *n* = 1083), 46.9% remained viraemic through follow‐up, 91.3% of which was high‐level viraemia. One‐fifth (20.8%) of visit‐pairs exhibiting persistent high‐level viraemia self‐reported antiretroviral therapy (ART) use for ≥12 months. Prevalence of persistent high‐level viraemia varied substantially across communities and was significantly elevated among young persons aged 15–29 years (vs. 40‐ to 49‐year‐olds; adjusted risk ratio [adjRR] = 2.96; 95% confidence interval [95% CI]: 2.21–3.96), males (vs. females; adjRR = 2.40, 95% CI: 1.87–3.07), persons reporting inconsistent condom use with non‐marital/casual partners (vs. persons with marital/permanent partners only; adjRR = 1.38, 95% CI: 1.10–1.74) and persons reporting hazardous alcohol use (adjRR = 1.09, 95% CI: 1.03–1.16). The prevalence of persistent high‐level viraemia was highest among males <30 years (32.0%).

**Conclusions:**

Following universal ART provision, most persons living with HIV in south‐central Uganda are durably suppressed. Among persons exhibiting any viraemia, nearly half exhibited high‐level viraemia for ≥12 months and reported higher‐risk behaviours associated with onward HIV transmission. Intensified efforts linking individuals to HIV treatment services could accelerate momentum towards HIV epidemic control.

## 1 INTRODUCTION

Universal Test and Treat (UTT) signalled a global paradigm shift in HIV control efforts through expanded antiretroviral therapy (ART) eligibility. Randomized trials in sub‐Saharan Africa demonstrated population‐level benefits of UTT implementation, with increased population viral load suppression (VLS) preceding significant HIV incidence declines [1, 2, 3, 4]. UTT strategies have rapidly expanded treatment coverage in high‐burden settings like Uganda, where the proportion of persons living with HIV on ART increased four‐fold over the last decade [5]. In some areas, ART use is approaching or has exceeded ambitious universal treatment coverage targets (≥90%) for HIV elimination [6, 7].

Disparities in HIV treatment outcomes throughout Africa, nevertheless, suggest that person‐level and place‐based factors can modify care engagement and ART adherence, attenuating the effectiveness of epidemic control strategies like UTT. Four seminal UTT trials in sub‐Saharan Africa reported suboptimal linkage to ART in specific populations (i.e. males, youth, migrants), and none of the UTT arms achieved globally established targets for population VLS [1–3, 8]. UTT implementation alone may, thus, be insufficient to close gaps along the HIV care continuum, especially for populations underserved by the existing landscape of HIV services [9, 10].

Furthermore, available evidence on longitudinal virologic outcomes is derived primarily from clinically engaged populations, who are distinct from care‐disengaged or treatment‐inexperienced persons. Population‐based estimates offer a more comprehensive assessment of progress towards HIV epidemic control, but population‐level studies examining durable VLS, or virologic control sustained over a period of time, are limited [11, 12, 13, 14]. Only two of these studies have been conducted in generalized HIV epidemic settings, one of which was implemented in 32 communities in Kenya and Uganda—reporting high rates of durable VLS (∼95%) among ART‐experienced persons during UTT rollout [12]. Likewise, another study was implemented in four hyperendemic Lake Victoria fishing communities [14], with demographic and sexual behaviour profiles atypical of most African communities [15, 16]—observing moderate levels of persistent viraemia amidst large increases in durable VLS during UTT scale‐up. Importantly, neither study distinguished high‐level viraemia (≥1000 HIV RNA copies/ml) from low‐level viraemia (200–999 copies/ml) [14], which has been linked to subsequent virologic failure and HIV‐1 drug resistance [17, 18, 19].

Accordingly, we examined longitudinal patterns and correlates of durable VLS following the mass scale‐up of UTT in 40 communities in southern Uganda, including 36 rural agrarian and semi‐urban trading communities and four Lake Victoria fishing communities. Our analyses offer unique insights into the evolving dynamics of population‐level high‐ and low‐level HIV viraemia during a period of substantial HIV service expansion and the characteristics of sub‐populations who remain viraemic despite UTT rollout.

## 2 METHODS

### 2.1 Study design

Data were derived from the Rakai Community Cohort Study (RCCS)—an open, population‐based HIV surveillance cohort implemented across 40 communities in and around Rakai, Uganda [15]. Located in southern Uganda, the RCCS study area is characterized by a heterogeneous HIV epidemic, with HIV burdens varying substantially in magnitude across communities (inland communities: 9–26% prevalence; fishing communities: 38–43% prevalence) [15]. Bordering Tanzania to the South and Lake Victoria to the East, the region is also distinguished by high population mobility, including seasonal and erratic in‐ and out‐migration driven by the fishing economy and other local industries [16, 20].

Households in enumerated RCCS communities are censused biennially (every ∼12–18 months), and individuals aged 15–49 years residing in RCCS catchment areas for ≥6 months (≥1 month in fishing communities) are eligible to participate. Enumerators administer structured surveys measuring household characteristics, sexual behaviours and HIV service utilization. HIV status is determined using a validated algorithm of three rapid tests in the field and laboratory‐based confirmatory testing by enzyme immunoassay [21]. Viral load testing is performed on stored plasma using the Abbott RealTime HIV‐1 assay (Abbott Molecular, Inc., Des Plaines, IL).

For this study, inclusion was restricted to persons living with HIV contributing ≥2 study visits over three survey rounds: Round 17 (February 2015–September 2016), Round 18 (October 2016–May 2018) and Round 19 (June 2018–October 2020). The observation period coincided with various shifts in HIV service delivery in Uganda, most notably UTT scale‐up—beginning in the four Lake Victoria fishing communities in 2014, and later expanding to inland communities in December 2016 [14].

### 2.2 VLS measures

The unit of analysis was a visit‐pair, defined as two consecutive study visits (V*_i_* +V*_i+j_*) during the observation period, with participants contributing up to two visit‐pairs. Individuals with a missed visit at Round 18 contributed one visit‐pair to the analysis (index visit (V*_i_*): Round 17, follow‐up visit (V*_i+j_*): Round 19).

A VLS cutpoint of <200 HIV RNA copies/ml was selected to distinguish persons with potentially transmissible viraemia from those with no demonstrated risk of onward HIV transmission [22]. Four longitudinal virologic outcomes were identified within visit‐pairs: (1) durable VLS (<200 copies/ml across visits); (2) new/renewed VLS (<200 copies/ml at follow‐up only); (3) viral rebound (<200 copies/ml at index visit only); and (4) persistent viraemia (≥200 copies/ml across visits). Viraemia was stratified by two categories based on viral copy counts, per World Health Organization guidelines: low‐level viraemia (200–999 copies/ml) and high‐level viraemia (≥1000 copies/ml) [23].

### 2.3 Statistical analysis

Data were managed and analysed in Stata/IC 15.1 (StataCorp LLC, College Station, TX). First, the characteristics of individuals included in the analytic cohort and those excluded from analysis were compared using Pearson's χ [2] (for categorical variables) and Wilcoxon rank‐sum tests (for continuous variables). Probabilities of study inclusion were then estimated using multivariable logistic regression, modelling factors associated with study exclusion. Stabilized inverse probability selection weights were then constructed from these adjusted marginal probabilities and applied to subsequent descriptive analyses to mitigate potential attrition bias induced by excluding participants with <2 study visits.

Unweighted and weighted prevalence estimates for visit‐pair‐level virologic outcomes were then calculated and compared across demographic factors. Sensitivity analyses employing alternative VLS cutpoints (<50, <400 and <1000 copies/ml) were conducted to examine whether VLS prevalence estimates varied across thresholds. Among persons with three study visits, longitudinal VLS patterns were summarized descriptively, characterizing 5‐year viral load trajectories during UTT scale‐up.

Next, within each visit‐pair, the proportions of high‐level and low‐level viraemia at follow‐up were assessed conditional on virologic outcomes at the index visit. Among visit‐pairs exhibiting persistent high‐level viraemia, the fraction and characteristics of persons self‐reporting ART use for ≥12 calendar months (i.e. self‐reported current ART use at both index and follow‐up visits) were estimated.

To further evaluate temporal patterns in persistent high‐level viraemia during UTT scale‐up, prevalence estimates for visit‐pair‐level virologic outcomes were compared over calendar period. Regional‐ and community‐level prevalence estimates for persistent high‐level viraemia were ascertained by aggregating the cumulative proportion of visit‐pairs exhibiting persistent high‐level viraemia within 10 regions (∼2–8 communities) and 40 communities. Sex‐specific prevalence estimates of persistent high‐level viraemia were also calculated and compared over calendar period within individual regions.

Lastly, multivariable Poisson regression—overall and stratified by sex and community type, respectively—with generalized estimating equations, exchangeable covariance matrices and robust standard errors modelled individual‐level factors associated with persistent high‐level viraemia, relative to sustained or new/renewed low‐level viraemia or suppression (<1000 copies/ml across visits or at follow‐up only). Given the elevated risks associated with onward HIV transmission among persons exhibiting high‐level viraemia [22], sustained or new/renewed VLS or low‐level viraemia served as the reference category in analysis. Assessed predictors (Table 1) included demographic (age, sex, marital status, education, occupation, household wealth [24], migration, community type) and behavioural factors (number of past‐year sexual partnerships, condom use consistency across sexual partner types [25], transactional sex, hazardous alcohol use [measured from an aggregated count of eight alcohol use consequences [26]] and intimate partner violence perpetration or victimization [adapted from the Revised Conflicts Tactics Scale [27]]). Within each visit‐pair, demographic factors were derived from a participant's index visit (V*_i_*), while behavioural factors were derived from the follow‐up visit (V*_i+j_*).

**TABLE 1:** Measurement and definition of independent variables from the Rakai Community Cohort Study (RCCS) included in the analysis.

Variable	Type	Definition
Educational attainment	Categorical	Level of schooling, complete or incomplete, from primary 1–7 (*Primary*); secondary 1–6 (*Secondary*); or technical/university, primary professional, O'level professional, primary/O'level apprenticeship or A'level apprenticeship (*Technical/University*). Participants who reported never attending school were classified as having *No Formal Education*.
Primary occupation	Categorical	Activities or work that keep participants busy on average day (for money or not), collapsed into the following categories: agriculture for home use/bartering/selling, housework in own home, housekeeping in someone else's home or home brewing (*Agriculture or Housework*); shopkeeping, trading/vending or hairdresser/salon owner (*Trading or Shopkeeping*); bar worker/owner, waitress/waiter/restaurant owner or sex worker (*Bar Work, Waitressing or Sex Work*); fishing (*Fishing‐Related Occupation*); or government/clerical/teaching, student, military/police, medical worker, casual labourer, construction, mechanic, transportation (trucker/boda boda), sports betting or unemployed (*Other*).
Religion	Categorical	Self‐reported religious identity, grouped by the following: Catholic, Protestant, Church of Uganda, Saved/Pentecostal (*Catholic/Christian*); *Muslim*; or *Other/None*.
Household wealth	Categorical	Enumerated from an aggregation of nine household possessions and dwelling characteristics (yes or no) and partitioned into quartiles (groups of four) standardized at each survey round, as described by Santelli et al [24].
Migration	Categorical	Self‐reported migration into an RCCS community from outside the study since the prior round (∼18 calendar months) (*In‐Migrants*) compared to individuals who did not migrate into the study area in the prior round (*Long‐Term Residents*).
Condom use	Categorical	Sometimes or never (*Inconsistent*) versus always (*Consistent*) using condoms in the past year with any non‐marital/casual partner (i.e. visitor, stranger, workmates/colleague, boss/work supervisor, employee or sugar daddy/mummy). Those reporting no partners or marital/permanent partners only (i.e. current/former spouses or long‐term partners) in the past year served as the referent group.
Transactional sex	Categorical	Giving and/or receiving money, gifts or favours in exchange for sex in the past year with any partner (yes or no).
Hazardous alcohol use	Count	Aggregated number of reported experiences in the past year following alcohol consumption, adapted from Miller et al. [26]: (1) unsteady gait, (2) fell over, (3) got angry, (4) got violent/into a fight, (5) difficulty speaking, (6) forgot events while drinking, (7) shaking hands the next morning, (8) felt ashamed. Participants reporting no alcohol consumption in the past year received scores of 0.
Any alcohol use consequences	Categorical	Reported any of the following consequences following alcohol use in the past year (any or none/no alcohol consumption), adapted from Miller et al. [26]: (1) unsteady gait, (2) fell over, (3) got angry, (4) got violent/into a fight, (5) difficulty speaking, (6) forgot events while drinking, (7) shaking hands the next morning, (8) felt ashamed.
Illicit drug use	Categorical	Used any of the following illicit substances in the past year (yes or no): marijuana, amphetamines, aero fuels (“glue”), amayirungi (“khat”), heroin or kuber.
Intimate partner violence^a^	Categorical	Reported perpetrating or experiencing any of the following with any partner in the past year (yes or no): (1) verbally abused/shouted; (2) pushed, slapped or held down; (3) punched with something that could injure; (4) kicked/dragged; (5) threatened with a weapon (knife, gun, fire, rope); (6) used threats to force someone to have sex; (7) physically forced to have sex; (8) forced to perform sexual acts against will. Items were derived from the Revised Conflicts Tactics Scale [27].

^a^ Variable first introduced during the Round 18 survey interval (October 2016–May 2018).

Finally, stabilized inverse probability of censoring weights were calculated to account for the differential inclusion of participants with three visits relative to individuals with only two visits. Inverse probability of selection and censoring weights were then multiplied together to calculate a final analytic weight for multivariable models. An optimal working correlation structure was identified from the quasi‐likelihood under the independence model criterion (QIC) [28]. Model‐wise deletion was used to address missing observations, accounting for <1% of all visit‐pairs.

### 2.4 Ethics

The Uganda Virus Research Institute Research and Ethics Committee and the Johns Hopkins University School of Medicine Institutional Review Board approved the study. Adults (≥18 years) and emancipated minors provided written informed consent prior to enrolment. Written assent and parental consent were obtained for unemancipated minors aged 15–17 years.

## 3 RESULTS

**FIGURE 1:**
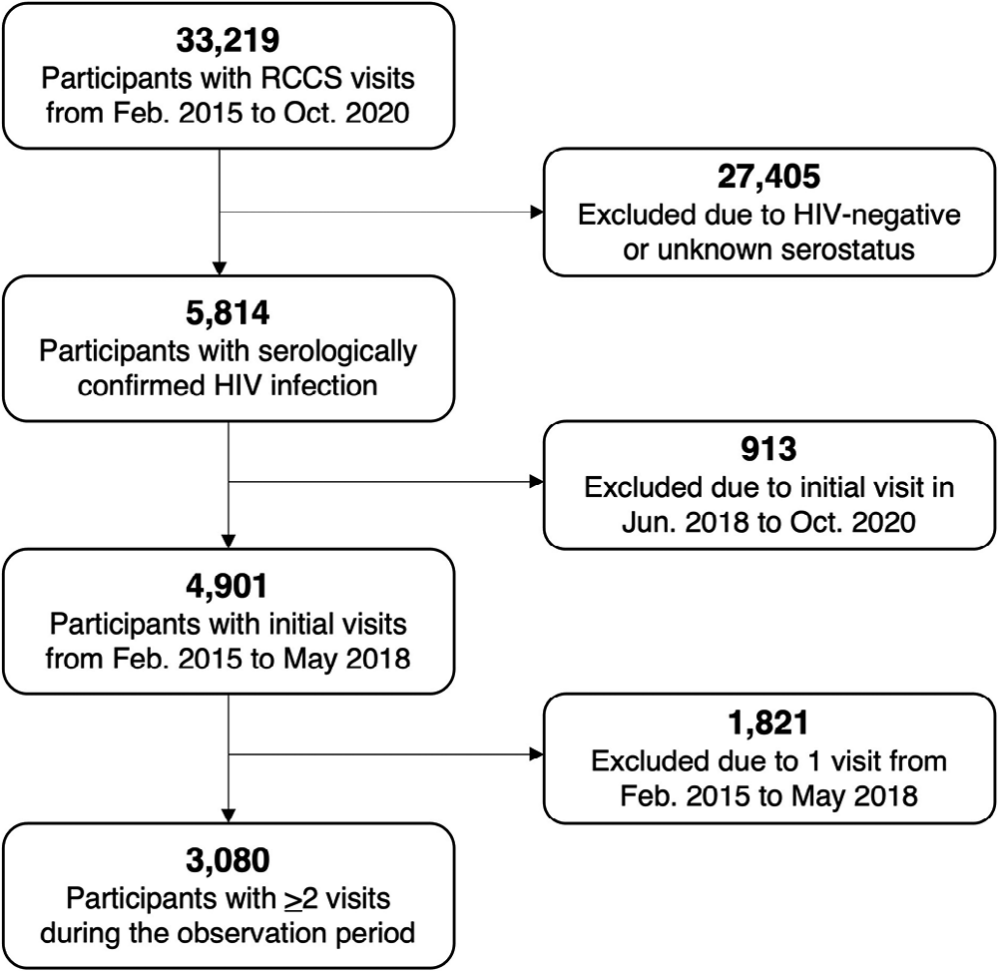
Flow chart of inclusion into the analytic cohort from the Rakai Community Cohort Study (RCCS).

From February 2015 to October 2020, 33,219 individuals participated in the RCCS, of whom 17.5% (*n* = 5814) were persons living with HIV (Figure 1). Persons whose index visits occurred at Round 19 (15.7%) were excluded. Of the remaining 4901 individuals eligible for study inclusion, 3080 (62.8%) had at least two study visits during the observation period and contributed 4604 visit‐pairs to the analysis. The remaining 1821 individuals, largely comprised of out‐migrants (60.9%), were excluded. At the index visit, 28.9% (*n* = 891) of the analytic cohort exhibited any viraemia, 79.8% of whom reported never having used ART.

Table 2 compares index survey visit characteristics of individuals included in the analytic cohort (median age: 34 years, 61.9% female) to those who were excluded (median age: 31 years, 63.7% female). Excluded individuals were significantly (*p*<0.05) younger, wealthier, more mobile and more likely to exhibit HIV‐related risk behaviours (e.g. more sexual partners, hazardous alcohol use) relative to the analytic cohort. HIV outcomes, including self‐reported history of ART use (69.8% vs. 57.4%, *p*<0.001) and VLS (71.1% vs. 59.3%, *p*<0.001), were also superior in the analytic cohort.

**TABLE 2:** Descriptive sample statistics at the index visit comparing participants with ≥2 visits (analytic cohort) to those with 1 visit (lost to follow‐up) during the observation period—2015–2020.

Characteristics (*n*, %)	Total *N* = 4901	Lost to follow‐up *n* = 1821 (37.2%)	Analytic cohort *n* = 3080 (62.8%)	χ^2^ *p*‐value^a^
*Demographics*				
Index survey visit (calendar period)				<0.001
Feb. 2015–Sep. 2016	3606 (73.6)	1118 (61.4)	2488 (80.8)	
Oct. 2016–May 2018	1295 (26.4)	703 (38.6)	592 (19.2)	
Age, in years (*median*, *IQR*)^b^	33 (28–39)	31 (26–38)	34 (28–39)	<0.001
Age group				<0.001
15–29 years	1688 (34.4)	780 (42.8)	908 (29.5)	
30–39 years	2102 (42.9)	681 (37.4)	1421 (46.1)	
40–49 years	1111 (22.7)	360 (19.8)	751 (24.4)	
Sex				0.226
Male	1835 (37.4)	662 (36.3)	1173 (38.1)	
Female	3066 (62.6)	1159 (63.7)	1907 (61.9)	
Currently marital status				0.131
Never married	374 (7.6)	153 (8.4)	221 (7.2)	
Currently married	2855 (58.3)	1032 (56.7)	1823 (59.2)	
Previously married	1672 (34.1)	636 (34.9)	1036 (33.6)	
Educational attainment				0.990
No formal education	412 (8.4)	152 (8.4)	260 (8.5)	
Primary	3581 (73.1)	1329 (73.0)	2252 (73.1)	
Secondary	787 (16.0)	296 (16.2)	491 (15.9)	
Technical/university	121 (2.5)	44 (2.4)	77 (2.5)	
Primary occupation				<0.001
Agriculture or housework	1706 (34.8)	605 (33.2)	1101 (35.8)	
Trading or shopkeeping	1037 (21.2)	400 (22.0)	637 (20.7)	
Bar work, waitressing or sex work	468 (9.6)	207 (11.4)	261 (8.5)	
Fishing‐related occupation	768 (15.7)	249 (13.7)	519 (16.8)	
Other	922 (18.8)	360 (19.8)	562 (18.2)	
Religion				0.398
Catholic/Christian	4291 (87.6)	1587 (87.1)	2704 (87.8)	
Muslim	572 (11.7)	216 (11.9)	356 (11.6)	
Other or none	38 (0.8)	18 (1.0)	20 (0.6)	
Household wealth (quartile)				<0.001
Lowest	2015 (41.1)	684 (37.6)	1331 (43.2)	
Low‐middle	1046 (21.3)	392 (21.5)	654 (21.2)	
High‐middle	1097 (22.4)	427 (23.4)	670 (21.8)	
Highest	728 (14.9)	309 (17.0)	419 (13.6)	
*Missing*	*15 (0.3)*	*9 (0.5)*	*6 (0.2)*	
Migration				<0.001
Long‐term resident	3510 (71.6)	1056 (58.0)	2454 (79.7)	
In‐migrant	1389 (28.3)	763 (41.9)	626 (20.3)	
*Missing*	*2 (0.1)*	*2 (0.1)*	*n/a*	
Community type				<0.001
Agrarian	1457 (29.7)	524 (28.8)	933 (30.3)	
Trading	1160 (23.7)	509 (27.9)	651 (21.1)	
Fishing	2284 (46.6)	788 (43.3)	1496 (48.6)	
Behavioural^c^				
Number of sexual partners				0.002
0–1	3359 (68.5)	1196 (65.7)	2163 (70.2)	
≥2	1542 (31.5)	625 (34.4)	917 (29.8)	
Condom use				0.067
No partners or permanent partners only	3310 (67.5)	1193 (65.5)	2117 (68.8)	
Consistent use with casual partners	698 (14.2)	275 (15.1)	423 (13.7)	
Inconsistent use with casual partners	893 (18.2)	353 (19.4)	540 (17.5)	
Transactional sex				0.017
No	2381 (47.3)	821 (45.1)	1497 (48.6)	
Yes	2583 (52.7)	1000 (54.9)	1583 (51.4)	
Any alcohol use consequences				0.030
No	3655 (74.6)	1326 (72.8)	2329 (75.6)	
Yes	1246 (25.4)	495 (27.2)	751 (24.4)	
Illicit drug use				0.372
No	4655 (95.0)	1723 (94.6)	2932 (95.2)	
Yes	246 (5.0)	98 (5.4)	148 (4.8)	
HIV‐related				
ART use history (self‐reported)				<0.001
Never	1706 (34.8)	775 (42.6)	931 (30.2)	
Currently or previously	3195 (65.2)	1046 (57.4)	2149 (69.8)	
HIV RNA viral load, in copies/ml				
Geometric mean viral load (95% CI)^b^	7792 (7061–8599)	8054 (6941–9344)	7581 (6645–8648)	0.551
<1000 copies/ml	3450 (70.4)	1160 (63.7)	2290 (74.4)	<0.001
<400 copies/ml	3324 (67.8)	1106 (60.7)	2218 (72.0)	<0.001
<200 copies/ml	3268 (66.7)	1079 (59.3)	2189 (71.1)	<0.001
<50 copies/ml	3121 (63.7)	1011 (55.5)	2110 (68.5)	<0.001

^a^ *p*‐values calculated using Pearson's chi‐square tests of association, unless otherwise specified.

^b^ *p*‐value calculated using Wilcoxon rank‐sum tests comparing median values and interquartile ranges (IQR).

^c^ Behavioural factors measured in the past year.

### 3.1 Prevalence of longitudinal viral load outcomes

Table 3 presents unweighted and weighted prevalence of longitudinal virologic outcomes at the visit‐pair level. Most visit‐pairs exhibited durable VLS (72.4%) or achieved VLS at the subsequent follow‐up visit (13.3%). The prevalence of persistent viraemia was 11.8%, with few visit‐pairs exhibiting viral rebound (2.5%). These prevalence estimates were insensitive to higher VLS cutpoints (Table S1).

**TABLE 3:** Prevalence of longitudinal virologic outcomes at visit‐pair level, by age group, sex and community type (*N* = 4604)

	Durable VLS			New/renewed VLS			Viral rebound			Persistent viraemia		
Characteristics	*n*/*N*	Crude%	Weighted%		Crude%	Weighted%	*n*/*N*	Crude%	Weighted%	*n*/*N*	Crude%	Weighted%
Overall	3408/4604	74.0	72.4	575/4604	12.5	13.3	113/4604	2.5	2.5	508/4604	11.0	11.8
Index survey visit												
(calendar period)^a^												
Feb. 2015–Sep. 2016	1725/2488	69.3	68.4	377/2488	15.2	15.4	60/2488	2.4	2.5	326/2488	13.1	13.7
Oct. 2016–May 2018	1683/2116	79.5	76.6	198/2116	9.4	11.1	53/2116	2.5	2.6	182/2116	8.6	9.7
Age group^a^												
15–29 years	699/1170	59.7	58.0	217/1170	18.6	19.3	34/1170	2.9	3.0	220/1170	18.8	19.7
30–39 years	1630/2149	75.9	75.6	256/2149	11.9	12.3	56/2149	2.6	2.6	207/2149	9.6	9.5
40–49 years	1079/1285	84.0	83.6	102/1285	7.9	8.1	23/1285	1.8	1.9	81/1285	6.3	6.4
Sex^a^												
Male	1101/1709	64.4	63.1	277/1709	16.2	16.7	57/1709	3.3	3.4	274/1709	16.0	16.8
Female	2307/2895	79.7	77.7	298/2895	10.3	11.4	56/2895	1.9	2.0	234/2895	8.1	8.9
Community type^b^												
Inland	1831/2418	75.7	73.5	267/2418	11.0	12.0	56/2418	2.3	2.4	264/2418	10.9	12.1
Fishing	1577/2186	72.1	71.1	308/2186	14.1	14.8	57/2186	2.6	2.7	244/2186	11.2	11.4
Males by age group^a^												
15–29 years	137/330	41.5	40.0	74/330	22.4	22.6	17/330	5.2	5.3	102/330	30.9	32.0
30–39 years	561/838	66.9	66.9	135/838	16.1	16.4	25/838	3.0	2.9	117/838	14.0	13.8
40–49 years	403/541	74.5	74.2	68/541	12.6	12.7	15/541	2.8	2.9	55/541	10.1	10.2
Females by age group^a^												
15–29 years	562/840	66.9	64.7	143/840	17.0	18.1	17/840	2.0	2.1	118/840	14.1	15.2
30–39 years	1069/1311	81.5	81.2	121/1311	9.2	9.7	31/1311	2.4	2.4	90/1311	6.9	6.7
40–49 years	676/744	90.8	90.5	34/744	4.6	4.7	8/744	1.1	1.1	26/744	3.5	3.7

*Notes*: “VLS” stands for HIV RNA viral load suppression, defined using an HIV RNA cutpoint of <200 copies/ml. “Crude. %” represents the unweighted prevalence of each viral load outcome at visit‐pair level. “Weighted %” represents the prevalence of each viral load outcome at visit‐pair level, corrected using stabilized inverse probability of selection weights.

^a^ Significant at the *p* < 0.001 level, based on Pearson's chi‐square tests of association using unweighted estimates.

^b^ Significant at the *p* < 0.01 level, based on Pearson's chi‐square tests of association using unweighted estimates.

Adults aged 40–49 years (83.6%) and females (77.7%) were significantly more likely to exhibit durable VLS (*p*<0.001) compared to young persons aged 15–29 years (58.0%) and males (58.0%), respectively. The prevalence of durable VLS was comparable in inland (73.5%) and fishing (71.1%) communities. Of note, young males (41.5%) and females (66.9%) exhibited significantly (*p*<0.001) lower fractions of durable VLS relative to older males and females (74.2% and 90.5%), respectively. Persistent viraemia was twice as common in young males (32.0%) relative to other age groups, irrespective of sex.

**FIGURE 2:**
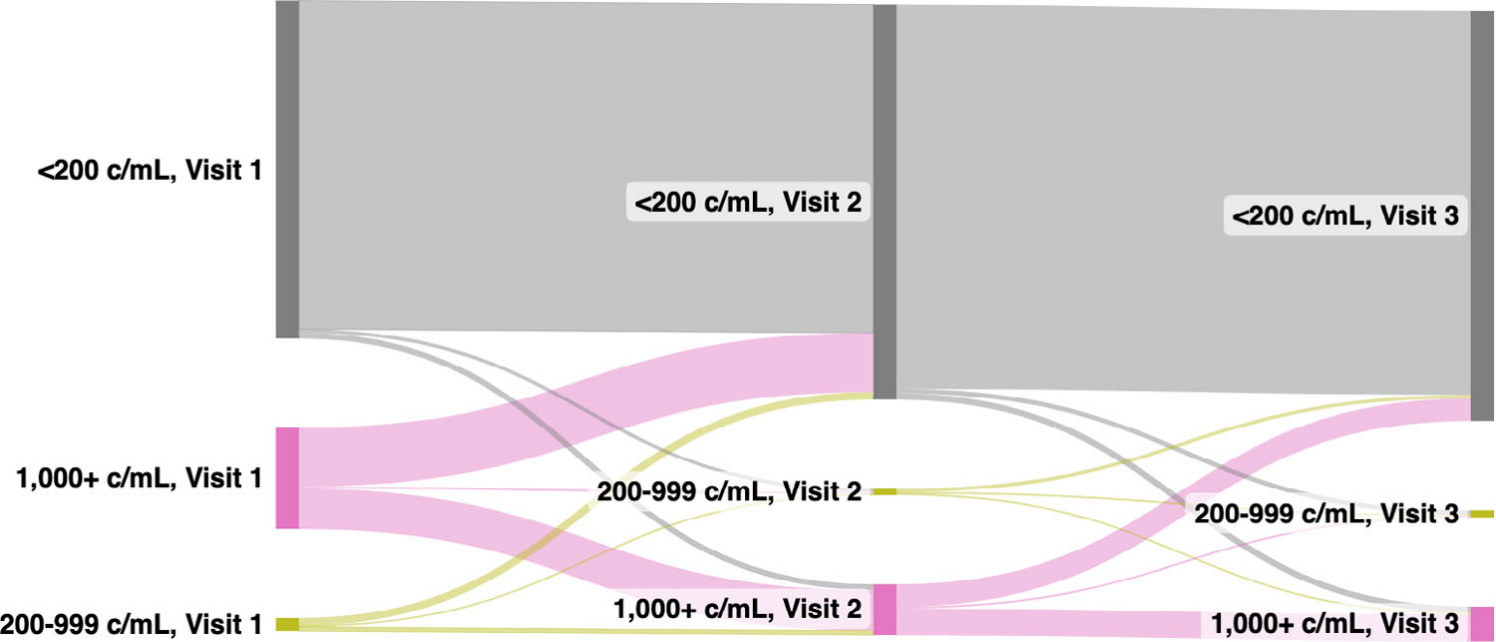
Sankey diagram of person‐level viral load trajectories among participants contributing three visits (two visit‐pairs) to the analytic cohort (*N* = 1524). Abbreviation: c/ml, HIV RNA copies/ml.

Among participants with three visits (*n* = 1524), a majority maintained VLS throughout (70.9%) or achieved VLS during (18.6%) the analysis period (Figure 2). Few participants exhibited persistent high‐ or low‐level viraemia (5.9%), high‐ or low‐level viral rebound (2.6%) or any intermittent viraemia (1.9%).

### 3.2 Dynamics of persistent high‐ and low‐level viraemia

**FIGURE 3:**
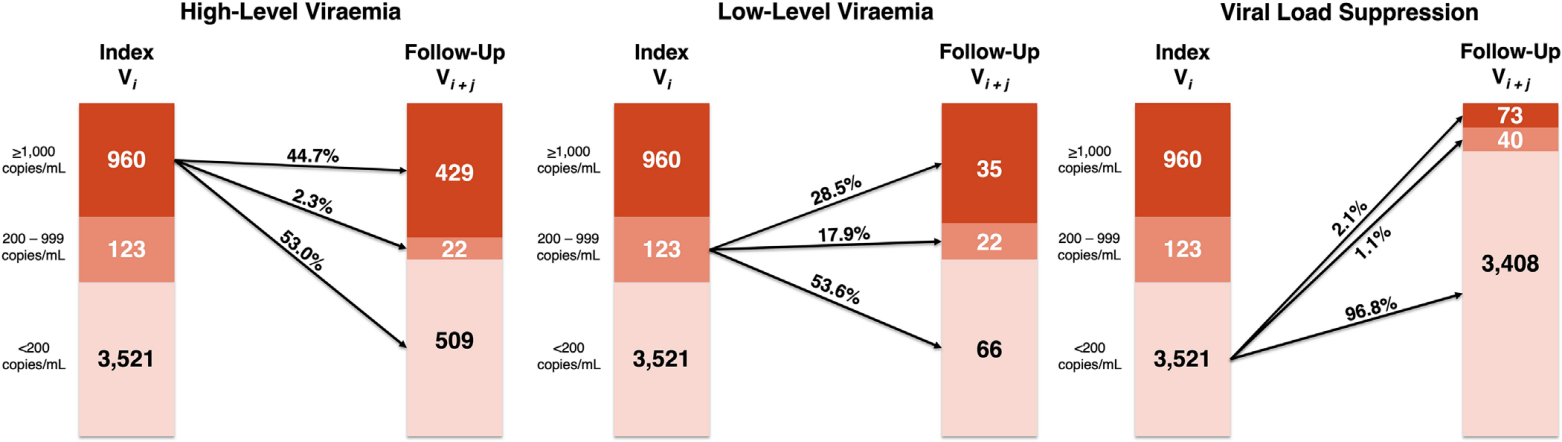
Conditional proportions of high‐ and low‐level HIV viraemia at follow‐up in the visit‐pair (*N* = 4604).

Figure 3 illustrates conditional proportions of high‐ and low‐level viraemia at follow‐up given viral load outcomes at the index visit. Among visit‐pairs exhibiting any viraemia at the index visit, nearly half (46.9%, *n* = 508/1083) remained viraemic through follow‐up. A substantially greater fraction of this viraemia was high‐level (91.3%, *n* = 464/508) as opposed to low‐level viraemia.

Individuals with high‐level viraemia at their index visits (*n* = 960) were slightly more likely to achieve VLS (53.0%) than sustain high‐level viraemia (44.7%) through follow‐up, with few (2.3%) progressing to low‐level viraemia. By comparison, individuals exhibiting VLS at their index visits (*n* = 3521) were substantially more likely to maintain VLS (96.8%) than experience rebounding low‐ (1.1%) or high‐level (2.1%) viraemia at follow‐up. Likewise, more than half of individuals with low‐level viraemia at their index visits (*n* = 123) achieved VLS at follow‐up (53.6%), but substantial fractions sustained low‐level viraemia (17.9%) or rebounded to high‐level viraemia (28.5%) at follow‐up—a rate 14 times greater than in individuals exhibiting VLS at their index visits. Conditional proportions of high‐ and low‐level viraemia were also comparable when estimated separately by calendar period (Figure S1) and community type (Figure S2), respectively. Among visit‐pairs with persistent high‐level viraemia (*n* = 429), 20.8% were in persons self‐reporting current ART use at their index and follow‐up visits (≥12 calendar months apart) (Table S2).

### 3.3 Temporal and geographic patterns of persistent high‐level viraemia

**FIGURE 4:**
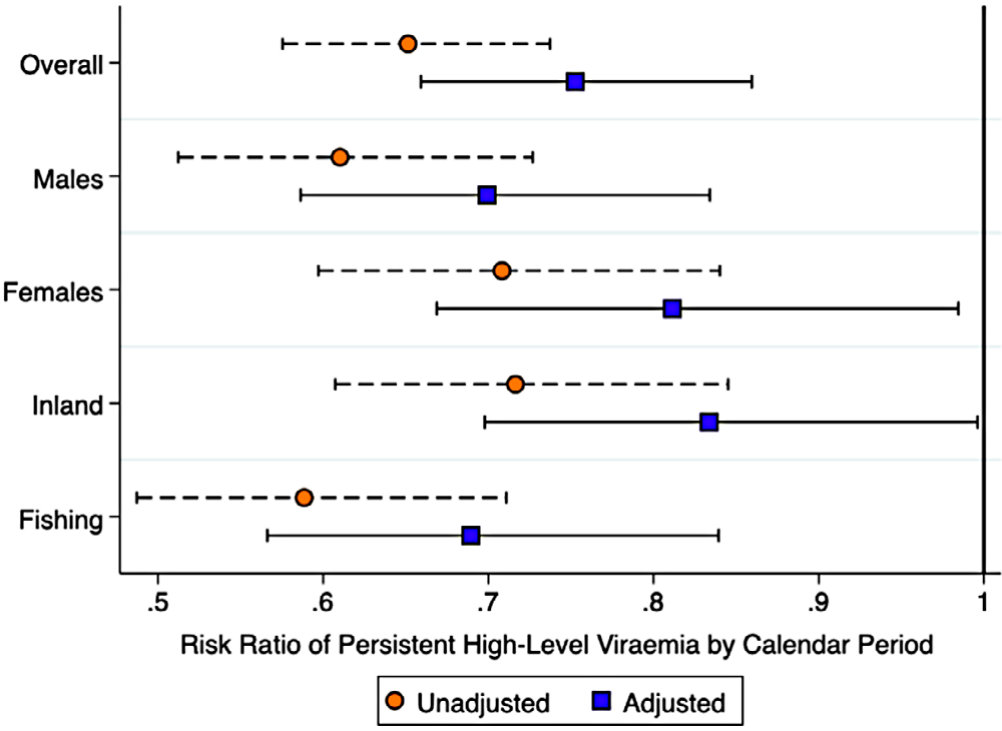
Weighted unadjusted and adjusted risk ratios of persistent high‐level viraemia (≥1000 copies/ml), relative to sustained or new low‐level viraemia or suppression, by calendar period—stratified by sex and community type. *Notes*: Risk ratios (RRs) and 95% confidence intervals (95% CI) were estimated from Poisson regression with generalized estimating equations, exchangeable covariance matrices, and robust standard errors. Error bars represent 95% CI for estimated risk ratios of persistent high‐level viraemia. Calendar period represents the survey interval in which a participant's index visit was observed (Oct. 2016–May 2018, *referent*: Feb. 2015–Sep. 2016). Sustained or new low‐level viraemia or suppression was defined as <1000 copies/ml across visits or at follow‐up only.

Significant increases (from 68.4% pre‐UTT scale‐up to 76.6% post‐UTT scale‐up) in the prevalence of durable VLS were accompanied by reductions in persistent high‐level viraemia (from 13.7% to 9.7%, adjusted risk ratio [adjRR] = 0.75, 95% confidence interval [95% CI] 0.66–0.86) (Table 3 and Figure 4). Significant reductions in persistent high‐level viraemia over calendar period were also observed across population strata (males: adjRR = 0.70, 95% CI 0.59–0.83; females: adjRR = 0.81, 95% CI 0.67–0.98) and community types (inland: adjRR = 0.83, 95% CI 0.70–1.00; fishing: adjRR = 0.69, 95% CI 0.57–0.84) (Figure 4).

**FIGURE 5:**
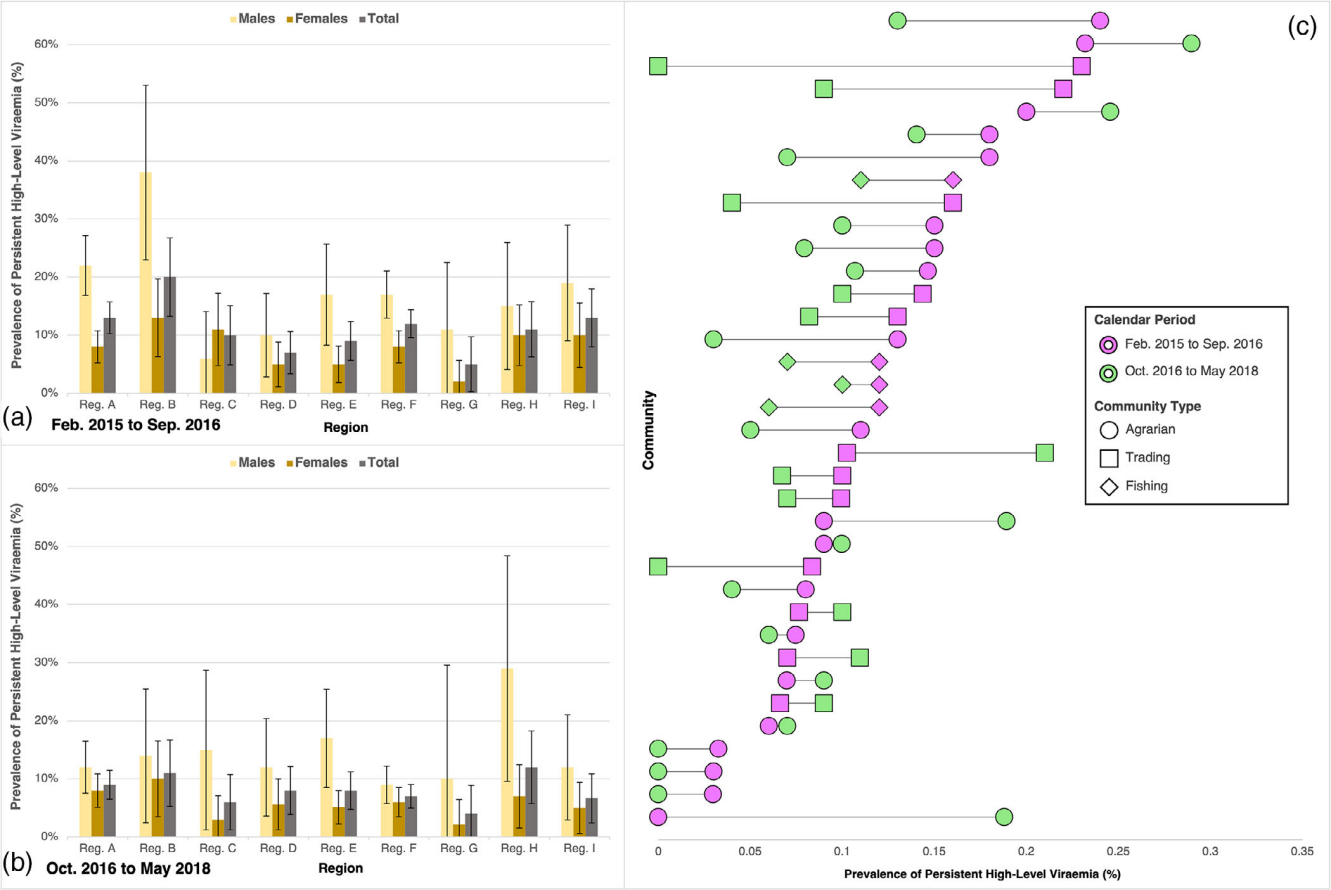
Weighted regional and community‐level prevalence of persistent high‐level viraemia (≥1000 copies/ml), by sex and calendar period. *Notes*: Error bars in panels A and B represent 95% confidence intervals (95% CI) for prevalence estimates of persistent high‐level viraemia. Bars in panel C represent the absolute difference in community‐level prevalence of persistent high‐level viraemia between survey rounds. Region names have been geomasked with letter identifiers.

Nevertheless, changes in the prevalence of persistent high‐level viraemia over time were not uniform, with some geographic areas exhibiting greater declines than others. Within regions, the prevalence of persistent high‐level viraemia declined in all but one region (Figure 5A,B), and the magnitude of these declines varied across regions (Table S3). Community‐level analyses revealed heterogeneities in the magnitudes of decline in persistent high‐level viraemia over calendar period (Figure 5C and Table S4). Across communities, however, observed declines in persistent high‐level viraemia were generally not statistically significant (Table S4), and community‐level variations in the magnitude of these declines were unexplained by differences in community type (Figure 5C).

### 3.4 Individual‐level predictors of persistent high‐level viraemia

Table 4 presents unweighted and weighted associations for persistent high‐level viraemia, relative to sustained or new/renewed VLS. Overall, males were more than twice as likely as females to exhibit persistent high‐level viraemia (adjRR = 2.40, 95% CI 1.87–3.07). Relative to persons with no past‐year sexual partners or permanent partners only, persons reporting inconsistent condom use with casual partners exhibited significantly elevated risk of persistent high‐level viraemia (adjRR = 1.38, 95% CI 1.10–1.74). Secondary education (adjRR = 1.51, 95% CI 1.17–1.95), relative to no formal education, and hazardous alcohol use (adjRR = 1.09, 95% CI 1.03–1.16) were also significantly associated with persistent high‐level viraemia. In‐migrants were also substantially, but not significantly, more likely than long‐term residents to exhibit persistent high‐level viraemia (adjRR = 1.19, 95% CI 0.99–1.44). Weighted multivariable models stratified by sex and community type, respectively, yielded similar findings (Tables S5 and S6).

**TABLE 4:** Unweighted and weighted risk of persistent high‐level viraemia (≥1000 copies/ml) relative to sustained or new/renewed low‐level viraemia or suppression (*N* = 3028)

	Unweighted		Weighted	
Characteristics	RR (95% CI)	adjRR (95% CI)	RR (95% CI)	adjRR (95% CI)
*Demographics*				
Age group				
15–29 years	**3.08 (2.34–4.05)**	**2.81 (2.12–3.73)**	**3.12 (2.34–4.15)**	**2.96 (2.21–3.96)**
30–39 years	**1.62 (1.25–2.11)**	**1.57 (1.21–2.03)**	**1.65 (1.26–2.17)**	**1.63 (1.24–2.15)**
40–49 years	*Ref*.	*Ref*.	*Ref*.	*Ref*.
Sex				
Male	**2.26 (1.84–2.77)**	**2.57 (2.02–3.27)**	**2.04 (1.63–2.53)**	**2.40 (1.87–3.07)**
Female	*Ref*.	*Ref*.	*Ref*.	*Ref*.
Currently marital status				
Never married	**1.82 (1.37–2.42)**	**1.34 (1.02–1.79)**	**1.78 (1.34–2.37)**	1.28 (0.96–1.70)
Currently married	*Ref*.	*Ref*.	*Ref*.	*Ref*.
Previously married	0.94 (0.77–1.15)	1.02 (0.84–1.25)	0.97 (0.78–1.22)	1.04 (0.84–1.29)
Completed education				
No formal education	0.78 (0.53–1.15)	0.91 (0.62–1.32)	0.84 (0.57–1.25)	1.02 (0.70–1.50)
Primary	*Ref*.	*Ref*.	*Ref*.	*Ref*.
Secondary	**1.31 (1.02–1.68)**	**1.36 (1.07–1.74)**	**1.43 (1.09–1.88)**	**1.51 (1.17–1.95)**
Technical/university	0.74 (0.34–1.62)	1.02 (0.48–2.18)	0.70 (0.31–1.55)	1.00 (0.45–2.23)
Primary occupation				
Agriculture or housework	*Ref*.	*Ref*.	*Ref*.	*Ref*.
Trading or shopkeeping	1.10 (0.88–1.38)	0.99 (0.79–1.24)	1.16 (0.84–1.33)	0.96 (0.77–1.21)
Bar/sex work or waitressing	0.75 (0.52–1.08)	0.80 (0.55–1.18)	0.69 (0.47–1.01)	0.75 (0.49–1.14)
Fishing‐related occupation	**1.83 (1.44–2.32)**	0.98 (0.75–1.29)	**1.58 (1.22–2.04)**	0.89 (0.67–1.18)
Other	1.23 (0.98–1.55)	0.96 (0.48–1.21)	1.14 (0.88–1.47)	0.93 (0.73–1.19)
Household wealth (quartile)				
Lowest	**1.43 (1.09–1.89)**	1.27 (0.96–1.69)	**1.36 (1.00–1.86)**	1.24 (0.91–1.70)
Low‐middle	**1.36 (1.02–1.81)**	1.14 (0.85–1.52)	1.27 (0.92–1.76)	1.09 (0.79–1.51)
High‐middle	**1.39 (1.07–1.83)**	1.29 (0.99–1.68)	1.33 (0.96–1.84)	1.22 (0.90–1.64)
Highest	*Ref*.	*Ref*.	*Ref*.	*Ref*.
Migration				
Long‐term resident	*Ref*.	*Ref*.	*Ref*.	*Ref*.
In‐migrant	**1.48 (1.23–1.79)**	**1.26 (1.04–1.52)**	**1.51 (1.24–1.82)**	1.19 (0.99–1.44)
*Behavioural*				
Number of sexual partners				
0–1	*Ref*.	*Ref*.	*Ref*.	*Ref*.
≥2	**1.47 (1.23–1.75)**	0.82 (0.67–1.01)	**1.42 (1.17–1.72)**	0.83 (0.67–1.03)
Condom use				
No partners or permanent partners only	*Ref*.	*Ref*.	*Ref*.	*Ref*.
Consistent use with casual partners	**1.44 (1.17–1.79)**	1.19 (0.95–1.50)	**1.51 (1.19–1.91)**	1.26 (0.99–1.61)
Inconsistent use with casual partners	**1.62 (1.34–1.98)**	**1.43 (1.16–1.77)**	**1.60 (1.29–1.99)**	**1.38 (1.10–1.74)**
Hazardous alcohol use	**1.15 (1.09–1.21)**	**1.07 (1.01–1.13)**	**1.16 (1.09–1.24)**	**1.09 (1.03–1.16)**
Intimate partner violence				
None	*Ref*.	*Ref*.	*Ref*.	*Ref*.
Any	**1.21 (1.03–1.44)**	1.03 (0.87–1.22)	**1.23 (1.04–1.46)**	1.05 (0.88–1.24)

*Note*: Risk ratios (RR) and 95% confidence intervals (95% CI) were estimated using Poisson regression with generalized estimating equations, exchangeable covariance matrices and robust standard errors. Multivariable models were adjusted for index survey interval and all covariates displayed in the columns presenting adjusted results. Sustained or new/renewed low‐level viraemia or suppression was defined as <1000 copies/ml across visits or at follow‐up only. **Bolded** values represent risk ratios of persistent HIV viraemia that were significantly different from the null value of 1 at the *p* < 0.05 level or below. Behavioural factors were measured in the past year.

## 4 DISCUSSION

This population‐based study in 36 moderate‐prevalence inland communities and four high‐prevalence fishing communities in southern Uganda demonstrated significant increases in durable HIV VLS coinciding with reductions in persistent high‐level viraemia during UTT scale‐up. Similar declines in persistent high‐level viraemia were observed by sex and age as well as across community types, suggesting UTT rollout conferred benefits across population strata and geographic settings. Despite the large fraction of persons exhibiting durable VLS or new/renewed suppression, persistent high‐level viraemia remained common in the presence of UTT. Persistent high‐level viraemia was also most frequently observed in specific groups—namely youth, males, in‐migrants and individuals with higher‐risk behaviours (i.e. inconsistent condom use with casual partners, hazardous alcohol use)—who experience substantial barriers along the HIV care continuum, from diagnosis to retention in care, throughout sub‐Saharan Africa [29, 30]. While UTT may accelerate progress towards HIV epidemic control goals, our findings reveal substantive disparities in longitudinal virologic outcomes amidst UTT scale‐up. These disparities are most pronounced in groups with the greatest potential for onward HIV transmission [31, 32, 33, 34].

A noteworthy fraction (∼20%) of persistent high‐level viraemia was observed in individuals self‐reporting ART use for ≥12 months, which could reflect unaddressed virologic failure and/or suboptimal ART adherence in this population. Factors associated with persistent viraemia among persons on ART reported elsewhere include HIV drug resistance, delayed switching to second‐ or third‐line ART regimens, viral load monitoring gaps and poor care quality [35, 36, 37]. Point‐of‐care viral load monitoring may reduce delays in detecting and responding to virologic failure among persons on ART exhibiting viraemia for >12 months [38]. Furthermore, persistently viraemic males were significantly more likely than females to have never initiated ART, which could reflect gendered patterns in anticipated/enacted HIV stigma, masculine norms surrounding care‐seeking and perceptions that health facilities are predominantly female‐oriented spaces [39, 40]. Collectively, these forces may dissuade males from initiating and continuing HIV care.

Despite observed increases in durable VLS coinciding with UTT scale‐up, declines in persistent high‐level viraemia were not consistent across communities, revealing geographic heterogeneities in the impact of UTT rollout. Although some of these differences could be explained by demographics, they were unexplained by community type alone, as declines varied even among communities of the same type. Other unmeasured factors (e.g. facility characteristics, HIV‐1 drug resistance) could be driving these observed geographic heterogeneities in persistent viraemia over time [41, 42]. Future investigation is needed to identify areas for potential health system interventions with the potential to accelerate the impact of UTT on population‐level VLS and HIV epidemic dynamics.

Persistent high‐level viraemia in this study was significantly more common among youth, males, in‐migrants and individuals with higher‐risk behavioural profiles (i.e. inconsistent condom use, hazardous alcohol use). These populations have been shown to shoulder disproportionate burdens of viraemia, both in this study setting and elsewhere [14, 43–45]. Of concern, over a third young males exhibited persistent high‐level viraemia—twice the rate observed in young females. Given that young males in this setting are highly mobile and report substantial barriers to HIV care linkage and retention (e.g. obtaining facility transfer forms) [20, 46, 47], expanding eligibility for less‐intensive differentiated service delivery (DSD) models (i.e. multi‐month ART dispensing, fast‐track drug refills) to some persistently viraemic persons and enhancing efficiencies in the facility transfer process are potential solutions to improving virologic outcomes in these groups. Enhanced linkage‐to‐care efforts and novel therapeutics like long‐acting ART may also optimize clinical outcomes in these populations.

This is the first study to characterize population‐level patterns and predictors of durable VLS and persistent high‐ and low‐level viraemia after UTT in both hyperendemic and moderate‐prevalence communities in sub‐Saharan Africa. Importantly, observed temporal patterns in population‐level viraemia and VLS may not be solely attributable to UTT scale‐up, given additional HIV service delivery innovations introduced during the observation period. These include the proliferation of less‐intensive DSD models (e.g. community ART distribution, multi‐month dispensing) and the transition to dolutegravir‐based regimens for first‐line ART, resulting in over two‐thirds of persons in the RCCS study area being initiated on or switched to an integrase strand‐transfer inhibitor by March 2020. Furthermore, consistent with findings from other African studies [48, 49, 50, 51], the final months of the observation period coincided with the onset of the COVID‐19 pandemic, which did not significantly impact VLS in this cohort [52].

Study findings are subject to several other limitations. First, study exclusion was substantial and associated with in‐ and out‐migration, which are known correlates of viraemia. Outcome estimates in the analytic cohort, therefore, likely underestimate the true prevalence of viraemia in the source population. Although an open cohort design and inverse probability weights helped address potential attrition biases induced by excluding individuals with only one study visit, this procedure attempts to balance the analytic cohort and the excluded population on measured *and* unobserved factors, which requires assumptions of exchangeability that are difficult to assess [53, 54]. Second, ART use and other behavioural factors were self‐reported and are, thus, susceptible to recall and acquiescence biases. Third, the present study did not include measures of HIV treatment adherence, use duration, interruptions, regimens or resistance—all of which are known antecedents of viraemia. Fourth, the interval between study visits was prolonged (≥12 calendar months), and VLS was ascertained only at the start and end of each interval. Additionally, most participants only contributed two visits to the analysis. Consequently, we lacked more repeated, frequent measurements of viraemia throughout visit intervals and could not discern longer‐term virologic trajectories for most participants. Fifth, individuals with three visits (two visit‐pairs) differed significantly from those with two visits (one visit‐pair) in the study population, notably on demographic and virologic outcomes (Table S7). Inverse probability weighting, nevertheless, helped mitigate potential biases induced by excluding unobserved visits from participants contributing two visits to the analytic cohort (Figure S3). Sixth, the study population was limited to individuals aged 15–49 years, so findings may not be transferable to adults aged 50+ years, whose viral load outcomes generally outperform those of younger populations [55, 56]. Lastly, while HIV epidemic characteristics of inland communities are comparable to settings outside the RCCS study area, findings from fishing communities may have more limited transferability to other contexts, given their unique demographic, behavioural and epidemiologic profiles [15, 16].

## 5 CONCLUSIONS

This study in 40 continuously surveilled communities in southern Uganda documented significant increases in durable VLS during UTT scale‐up. Nevertheless, noteworthy individual and geographic disparities in persistent high‐level viraemia coincided with population‐level gains in durable VLS. These gains in durable VLS, importantly, fall below UNAIDS suppression targets (86%) [7], indicating efforts must be intensified to achieve long‐term HIV epidemic control in Uganda. Furthermore, participants with any viraemia were equally as likely to achieve VLS or sustain viraemia, and those exhibiting low‐level viraemia initially were 14 times more likely than persons with initial VLS to rebound to high‐level viraemia. Youth, males, in‐migrants and individuals reporting higher‐risk behaviours exhibited significantly elevated risk of persistent high‐level viraemia during UTT scale‐up, suggesting that universal ART provision alone does not close gaps in virologic outcomes in populations at greatest risk for onward HIV transmission. To reach ambitious VLS targets, complementary treatment optimization interventions should be directed at populations who remain persistently viraemic in the presence of UTT. These include enhanced linkage to HIV care, expedited and more frequent viral load testing, long‐acting ART introduction, continued transition to dolutegravir‐based ART regimens, sustained geographic prioritization and streamlining facility transfer processes.

## COMPETING INTERESTS

The authors have no competing interests to disclose.

## AUTHORS’ CONTRIBUTIONS

JGR, LWC, VS, KBR, EUP, CEK, FN, GK, OR, AART, TCQ, JK, SJR and MKG conceptualized the present study and developed the methodology. RS, VS, RMG, AK, GN, FN, GK and JK oversaw data collection, with data management support from JS and AN. JGR, LWC, VS, GN, CEK, FN, GK, LJN, LAM, DK, JK, SJR and MKG acquired funding to support the present study. JGR led the data analysis, with input from LWC, VS, KBR, EUP, SJR and MKG. JGR prepared the first manuscript draft. All authors participated in data interpretation, revised the manuscript and had final responsibility for the decision to submit for publication.

## FUNDING

This study was supported by the National Institute of Allergy and Infectious Diseases (U01AI100031, U01AI075115, R01AI110324, R01AI128779, R01AI123002, R01AI143333, R01AI155080, T32AI102623); the National Institute of Mental Health (R01MH105313); the National Cancer Institute (75N91019D00024); and the U.S. President's Emergency Plan for AIDS Relief through the Centers for Disease Control and Prevention under the terms of NU2GGH00081. JGR and KBR were supported by the National Institute of Mental Health (F31MH126796, K01MH129226). EUP was supported by the National Institute on Drug Abuse (F31DA054849).

## DISCLAIMER

The contents of this manuscript are solely the responsibility of the authors and do not necessarily reflect the views or policies of the funding agencies, nor do mentions of trade names, commercial products or organizations imply endorsement by the U.S. Government.

## Supporting information


**Table S1**: Longitudinal virologic outcomes at visit‐pair level, by viral load suppression (VLS) cutpoints (*N* = 4,604).
**Figure S1**: Conditional proportions of HIV viraemia at follow‐up in the visit‐pair, by calendar period.
**Figure S2**: Conditional proportions of HIV viraemia at follow‐up in the visit‐pair, by community type.
**Table S2**: Visit‐pair‐level characteristics of participants exhibiting persistent high‐level viraemia, by self‐reported ART status.
**Table S3**: Weighted region‐level prevalence of persistent high‐level viraemia, by sex and calendar period.
**Table S4**: Weighted community‐level prevalence of persistent high‐level viraemia, by calendar period.
**Table S5**: Risk of persistent high‐level HIV viraemia (>1,000 copies/mL) relative to sustained or new/renewed low‐level viraemia or suppression, by sex.
**Table S6**: Risk of persistent high‐level HIV viraemia (>1,000 copies/mL) relative to sustained or new/renewed low‐level viraemia or suppression, by community type.
**Table S7**: Descriptive sample statistics at the index visit for participants contributing one visit‐pair (two visits) versus two visit‐pairs (three visits) to the analysis—2015 to 2020.
**Figure S3**: Box plots of stabilized inverse probability of selection and censoring weights, by number of visit‐pairs contributed to the analysis.

## Data Availability

De‐identified Rakai Community Cohort Study data can be provided to interested parties subject to the completion of the Rakai Health Sciences Program data request form and the signing of a Data Transfer Agreement. Inquiries should be directed to datarequests@rhsp.org.
