# Supplementary material for: Temporal dynamics and drivers of durable HIV viral load suppression and persistent high‐ and low‐level viraemia during Universal Test and Treat scale‐up in Uganda: a population‐based study
Source: J Int AIDS Soc. 2024 Feb 8;27(2):e26200. doi: 10.1002/jia2.26200 (PMC10853573; doi:10.1002/jia2.26200)
Supplement: Supplementary file 1 — Table S1: Longitudinal virologic outcomes at visit‐pair level, by viral load suppression (VLS) cutpoints (N = 4,604). Figure S1: Conditional proportions of HIV viraemia at follow‐up in the visit‐pair, by calendar period. Figure S2: Conditional proportions of HIV viraemia at follow‐up in the visit‐pair, by community type. Table S2: Visit‐pair‐level characteristics of participants exhibiting persistent high‐level viraemia, by self‐reported ART status. Table S3: Weighted region‐level prevalence of persistent high‐level viraemia, by sex and calendar period. Table S4: Weighted community‐level prevalence of persistent high‐level viraemia, by calendar period. Table S5: Risk of persistent high‐level HIV viraemia (>1,000 copies/mL) relative to sustained or new/renewed low‐level viraemia or suppression, by sex. Table S6: Risk of persistent high‐level HIV viraemia (>1,000 copies/mL) relative to sustained or new/renewed low‐level viraemia or suppression, by community type. Table S7: Descriptive sample statistics at the index visit for participants contributing one visit‐pair (two visits) versus two visit‐pairs (three visits) to the analysis—2015 to 2020. Figure S3: Box plots of stabilized inverse probability of selection and censoring weights, by number of visit‐pairs contributed to the analysis. [file JIA2-27-e26200-s001.docx]

**ELECTRONIC SUPPLEMENTARY MATERIAL**

**Temporal dynamics and drivers of durable HIV viral load suppression and persistent high- and low-level viraemia during Universal Test and Treat scale-up in Uganda: a population-based study**

Joseph G. Rosen, Robert Ssekubugu, Larry W. Chang, Victor Ssempijja, Ronald M. Galiwango, Joseph Ssekasanvu, Anthony Ndyanabo, Alice Kisakye, Gertrude Nakigozi, Katherine B. Rucinski, Eshan U. Patel, Caitlin E. Kennedy, Fred Nalugoda, Godfrey Kigozi, Oliver Ratmann, Lisa J. Nelson, Lisa A. Mills, Donna Kabatesi, Aaron A.R. Tobian, Thomas C. Quinn, Joseph Kagaayi, Steven J. Reynolds, M. Kate Grabowski

Table of Contents

[Table S1. Longitudinal virologic outcomes at visit-pair level, by viral load suppression (VLS) cutpoints (*N* = 4,604). 3](#_Toc153267887)

[Figure S1. Conditional proportions of HIV viraemia at follow-up in the visit-pair, by calendar period. 4](#_Toc153267888)

[Figure S2. Conditional proportions of HIV viraemia at follow-up in the visit-pair, by community type. 5](#_Toc153267889)

[Table S2. Visit-pair-level characteristics of participants exhibiting persistent high-level viraemia, by self-reported ART status. 6](#_Toc153267890)

[Table S3. Weighted region-level prevalence of persistent high-level viraemia, by sex and calendar period. 8](#_Toc153267891)

[Table S4. Weighted community-level prevalence of persistent high-level viraemia, by calendar period. 9](#_Toc153267892)

[Table S5. Risk of persistent high-level HIV viraemia (>1,000 copies/mL) relative to sustained or new/renewed low-level viraemia or suppression, by sex. 10](#_Toc153267893)

[Table S6. Risk of persistent high-level HIV viraemia (>1,000 copies/mL) relative to sustained or new/renewed low-level viraemia or suppression, by community type. 11](#_Toc153267894)

[Table S7. Descriptive sample statistics at the index visit for participants contributing one visit-pair (two visits) versus two visit-pairs (three visits) to the analysis—2015 to 2020. 12](#_Toc153267895)

[Figure S3. Box plots of stabilized inverse probability of selection and censoring weights, by number of visit-pairs contributed to the analysis. 14](#_Toc153267896)

# **Table S1.** Longitudinal virologic outcomes at visit-pair level, by viral load suppression (VLS) cutpoints (*N* = 4,604).

| **VLS Cutpoint** | **Durable VLS** | | |  | **New/Renewed VLS** | | |  | **Viral Rebound** | | |  | **Persistent Viraemia** | | |
| --- | --- | --- | --- | --- | --- | --- | --- | --- | --- | --- | --- | --- | --- | --- | --- |
|  | n/N | Crude  % | Weighted  % |  | n/N | Crude  % | Weighted  % |  | n/N | Crude  % | Weighted  % |  | n/N | Crude  % | Weighted  % |
| <1,000 copies/mL | 3,536/4,604 | 76.8 | 75.3 |  | 531/4,604 | 11.5 | 12.3 |  | 108/4,604 | 2.4 | 2.5 |  | 429/4,604 | 9.3 | 9.9 |
| <400 copies/mL | 3,449/4,604 | 74.9 | 73.4 |  | 566/4,604 | 12.3 | 13.1 |  | 112/4,604 | 2.4 | 2.5 |  | 477/4,604 | 10.4 | 11.1 |
| <200 copies/mL | 3,408/4,604 | 74.0 | 72.4 |  | 575/4,604 | 12.5 | 13.3 |  | 113/4,604 | 2.5 | 2.5 |  | 508/4,604 | 11.0 | 11.8 |
| <50 copies/mL | 2,978/4,604 | 64.7 | 63.1 |  | 647/4,604 | 14.1 | 14.9 |  | 333/4,604 | 7.2 | 7.1 |  | 646/4,604 | 14.0 | 14.9 |

*Notes*: “Crude. %” represents the unweighted prevalence of each viral load outcome at visit-pair level, by survey attribute. “Weighted %” represents the prevalence of each viral load outcome at visit-pair level, corrected using stabilized inverse probability of selection weights.

# **Figure S1**. Conditional proportions of HIV viraemia at follow-up in the visit-pair, by calendar period.


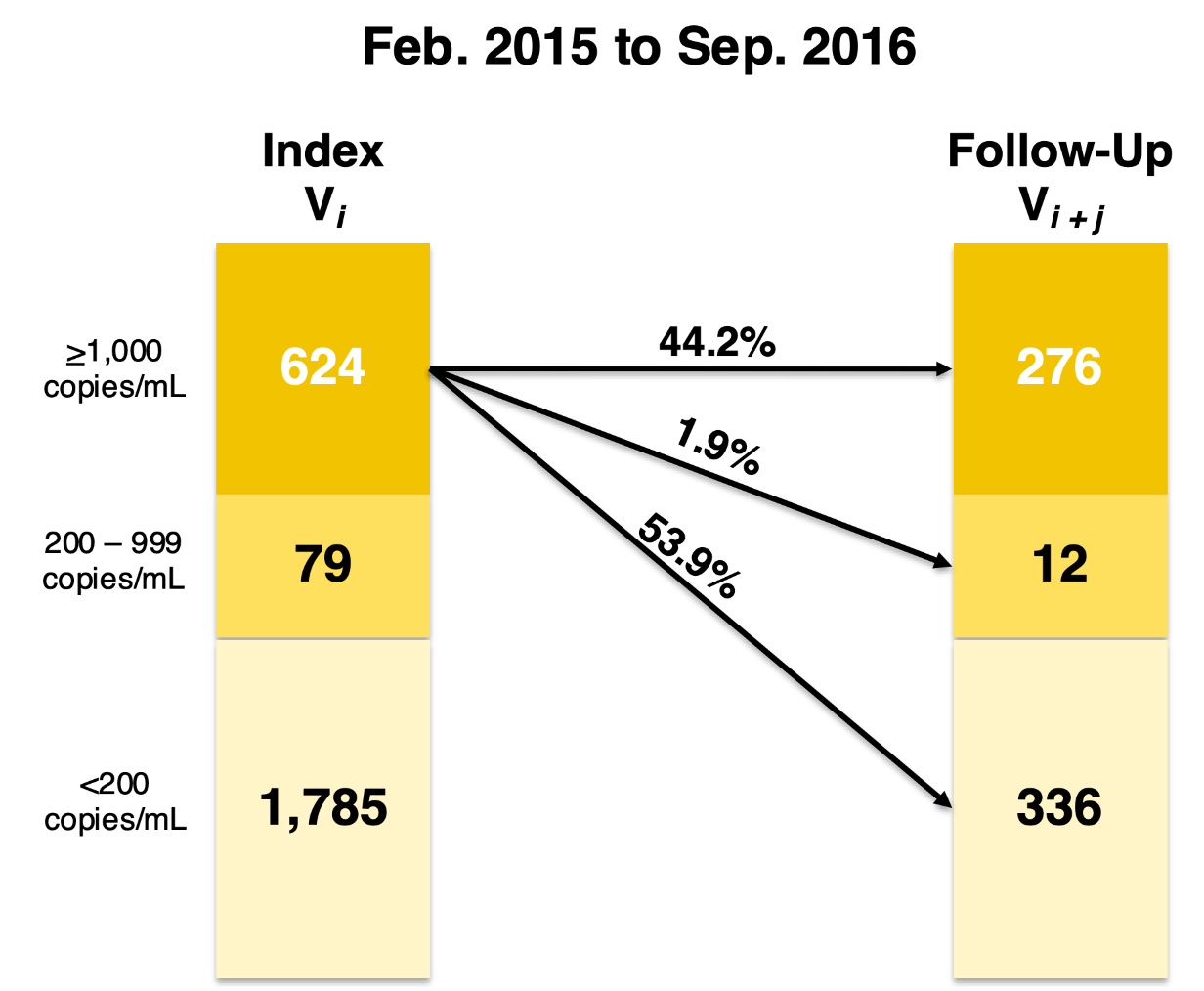

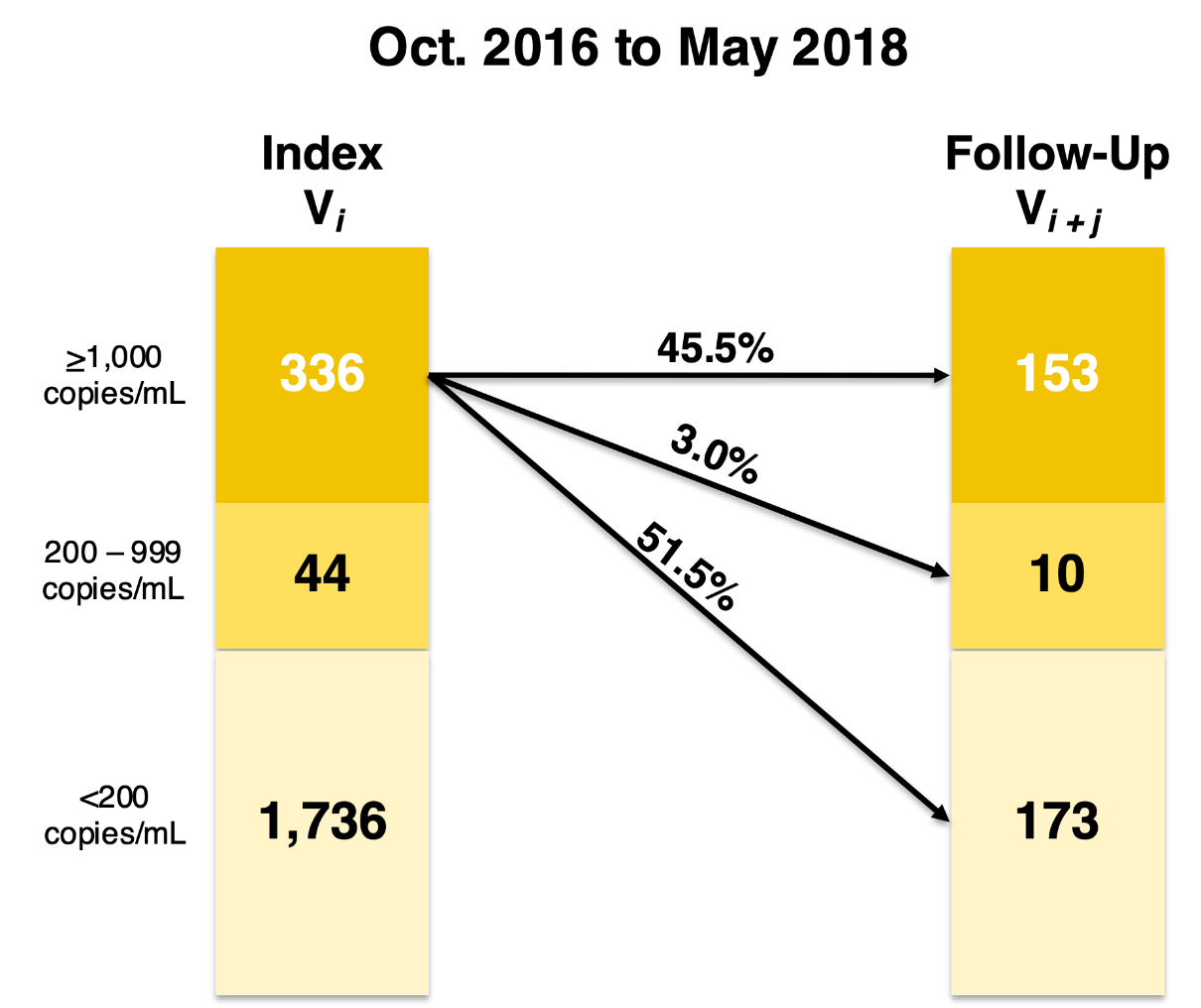


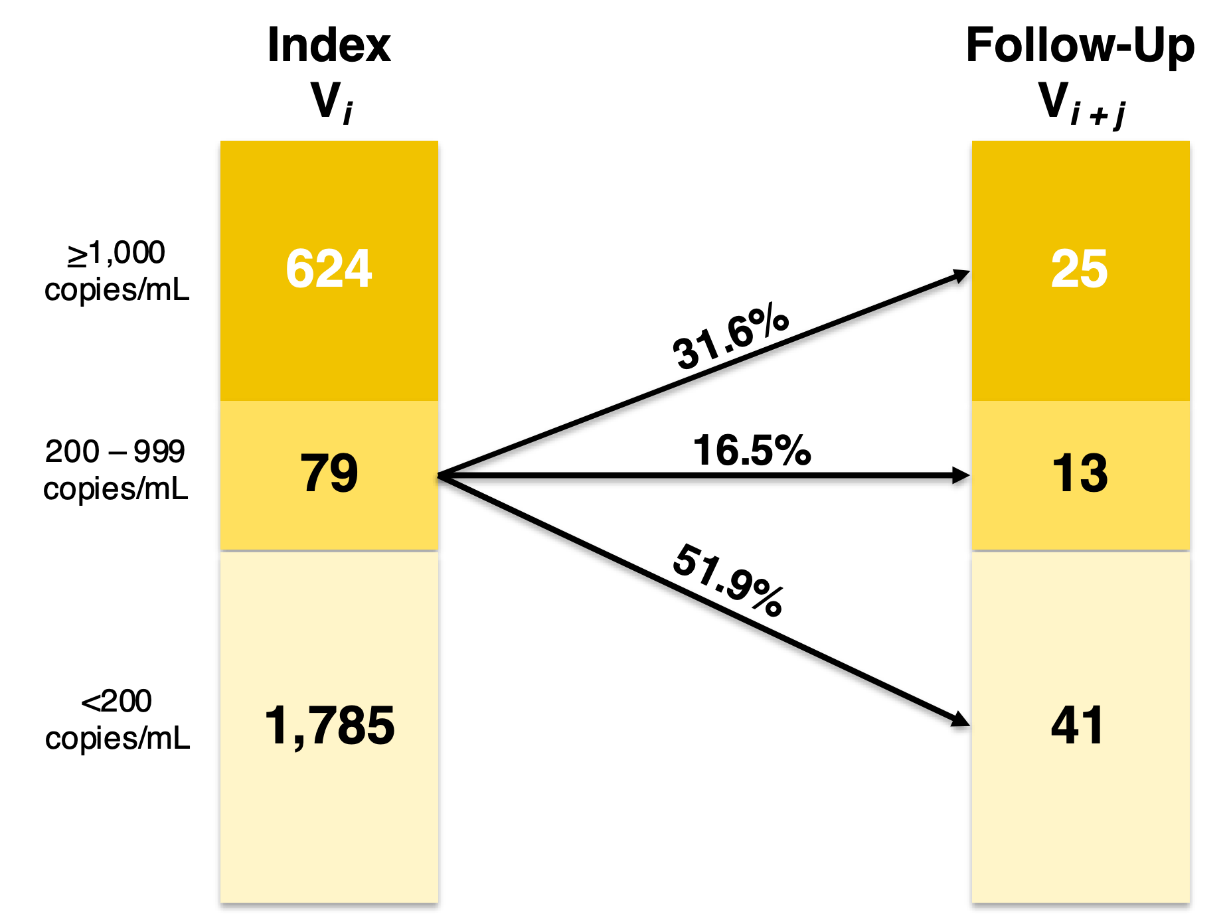

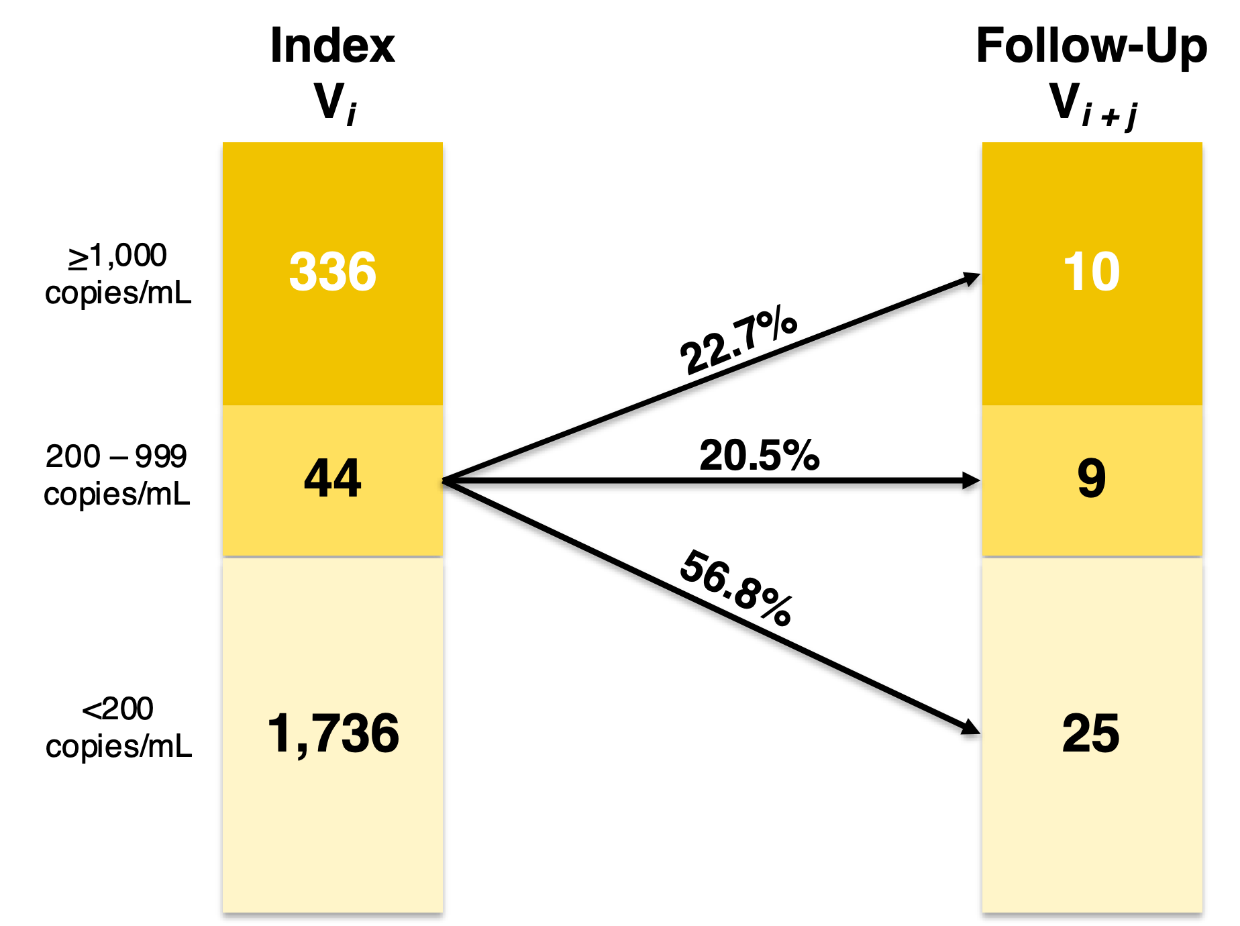


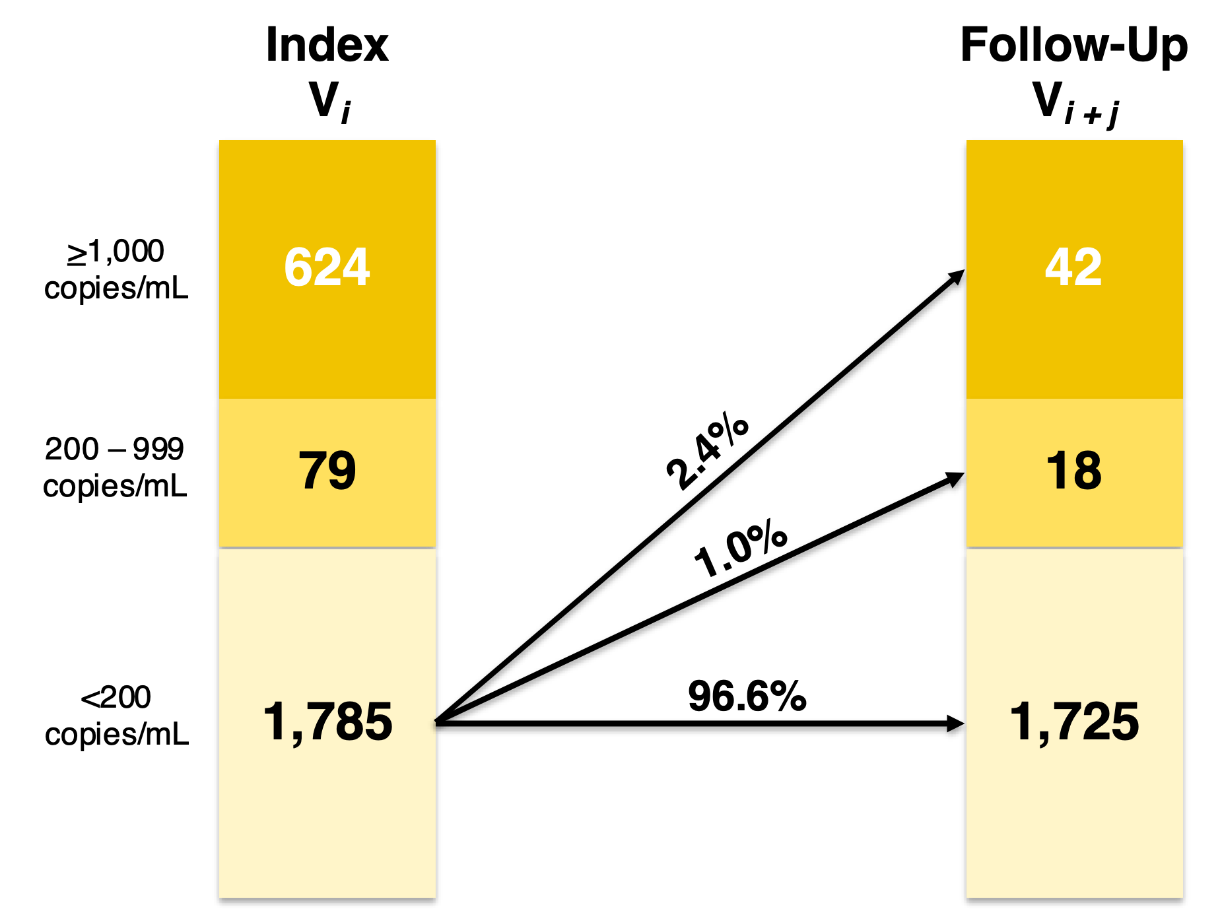

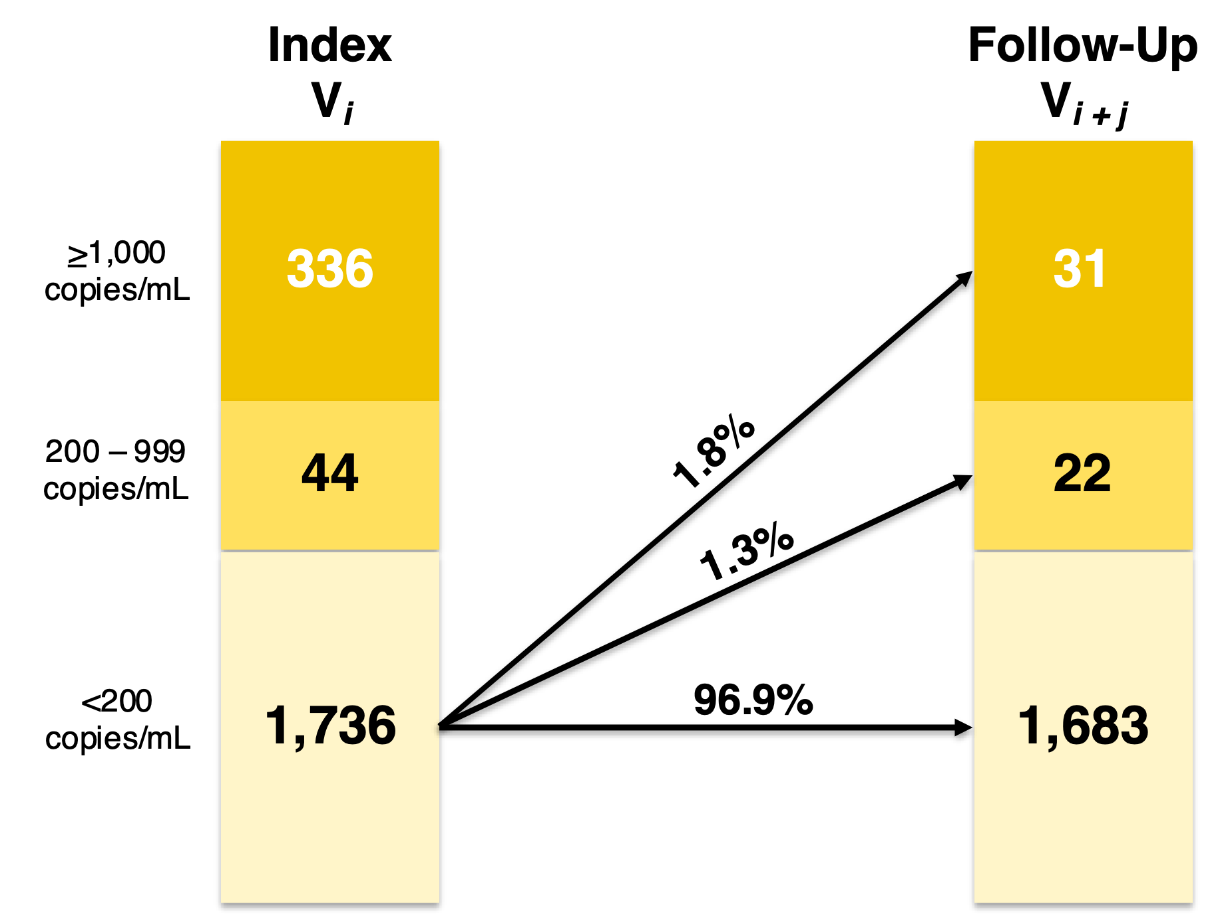


# **Figure S2**. Conditional proportions of HIV viraemia at follow-up in the visit-pair, by community type.


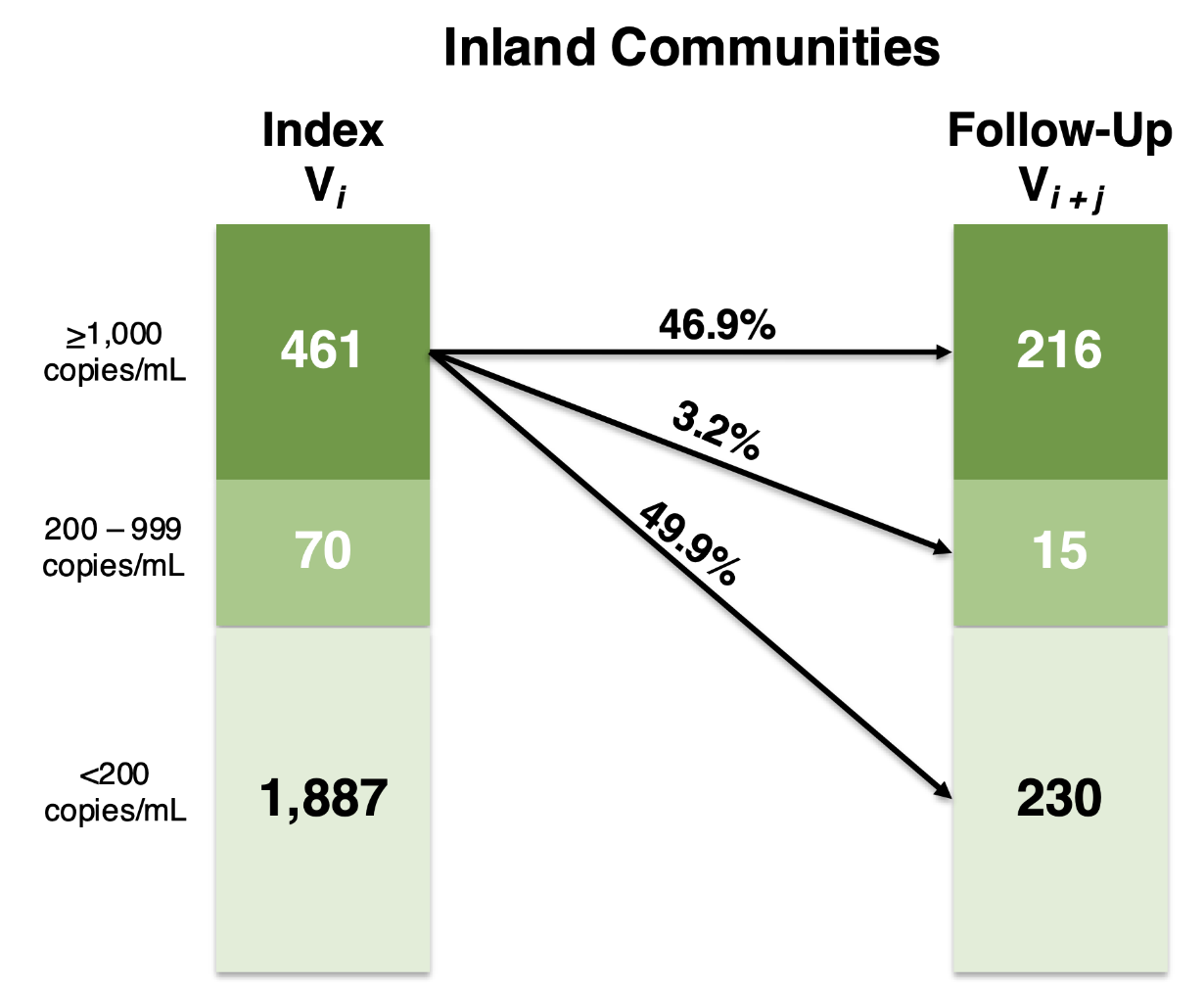

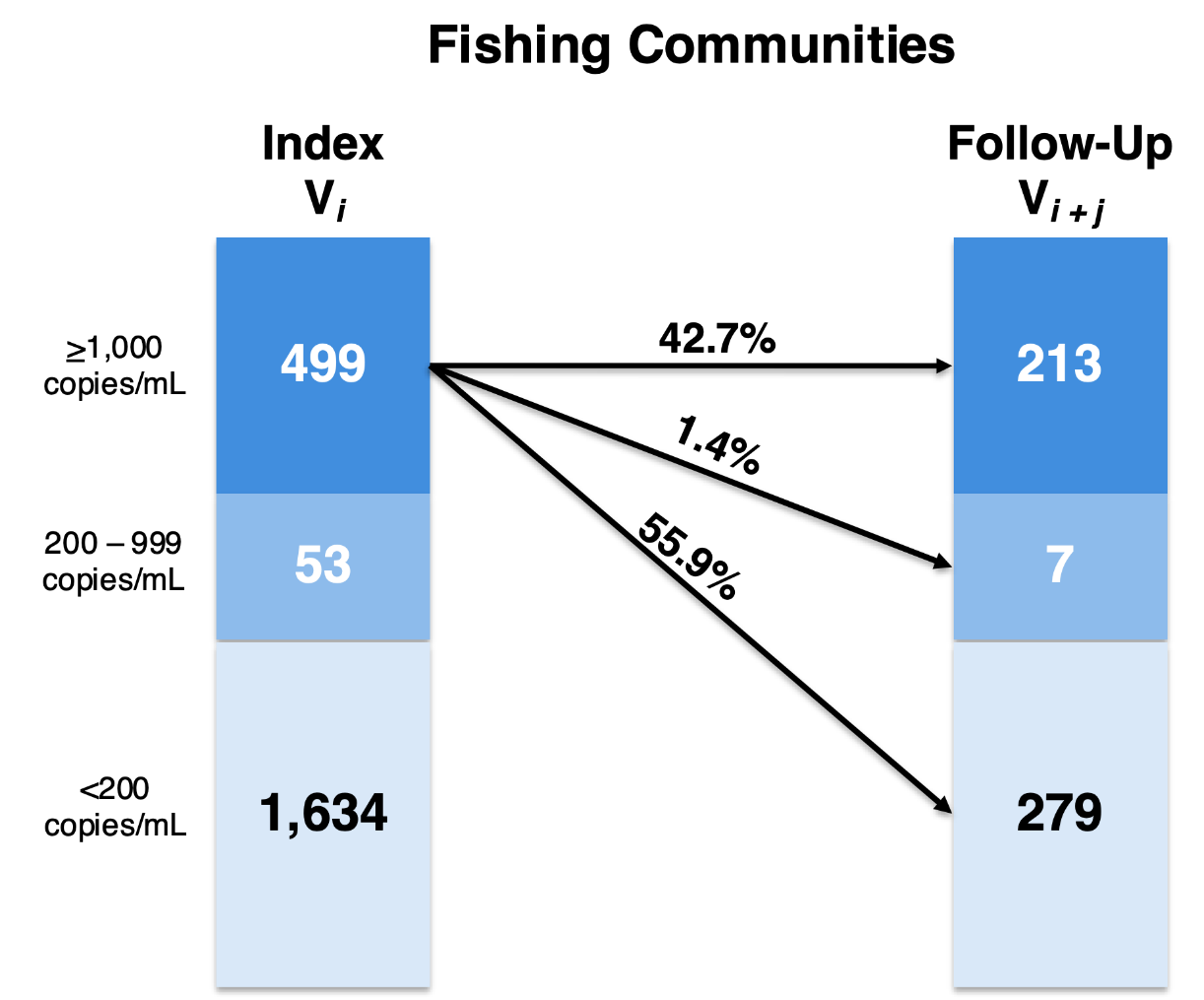


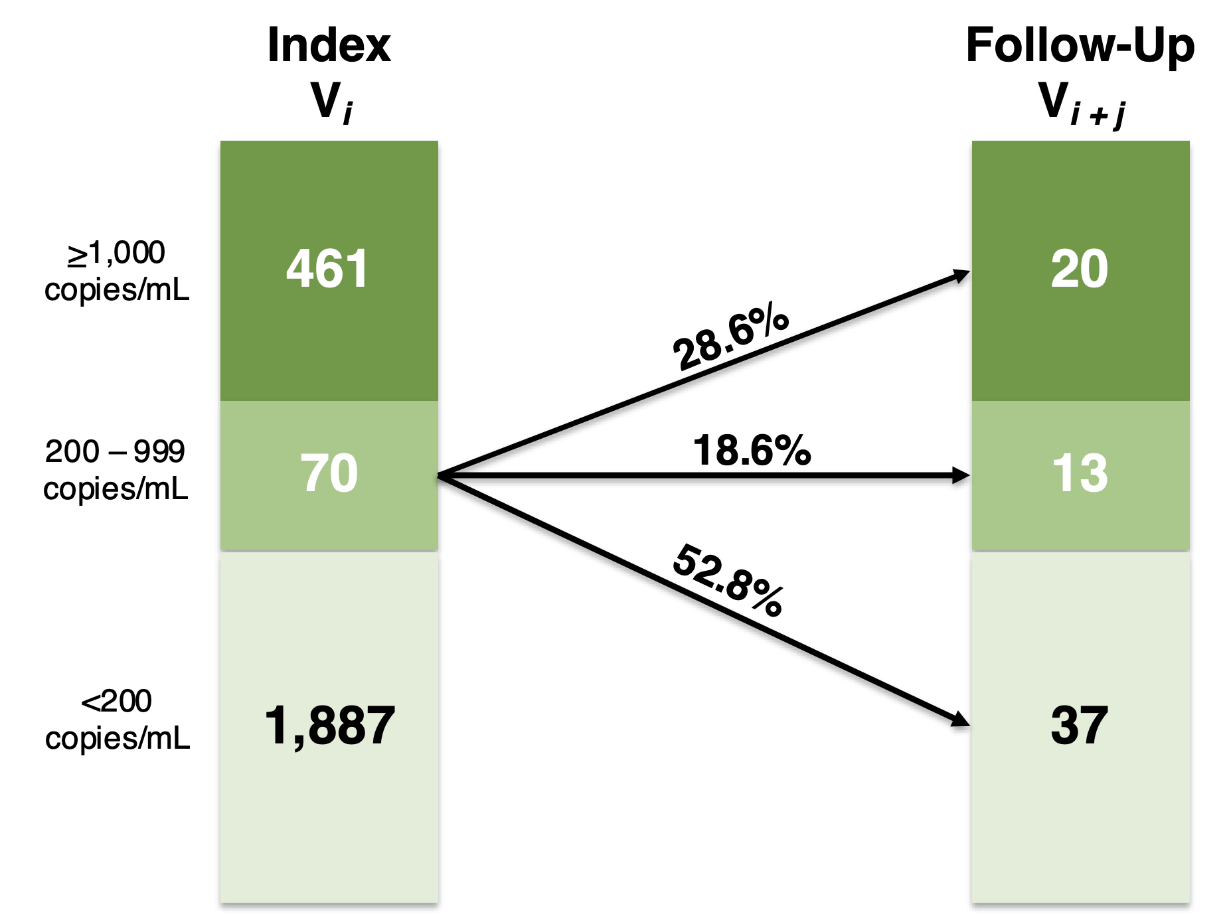

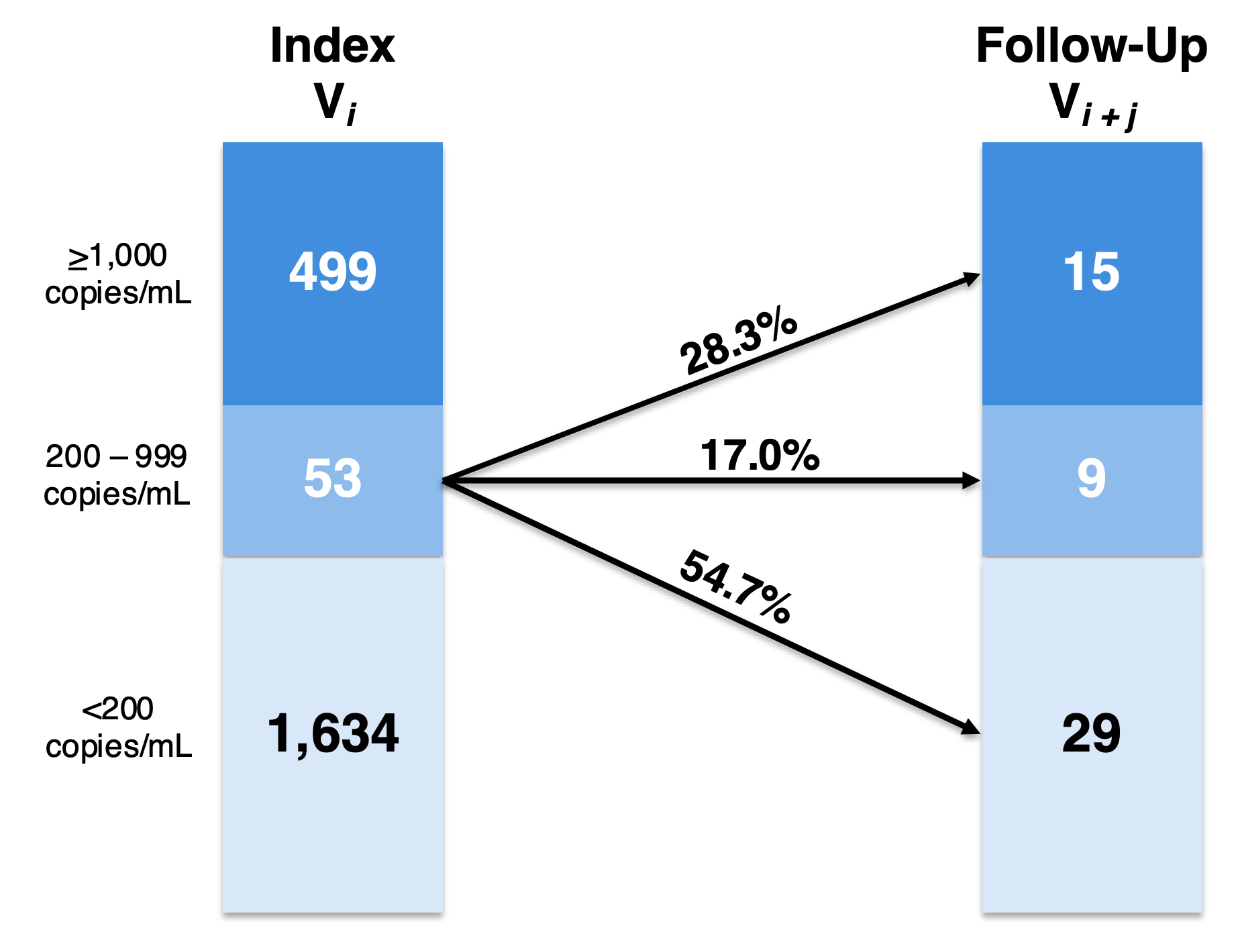


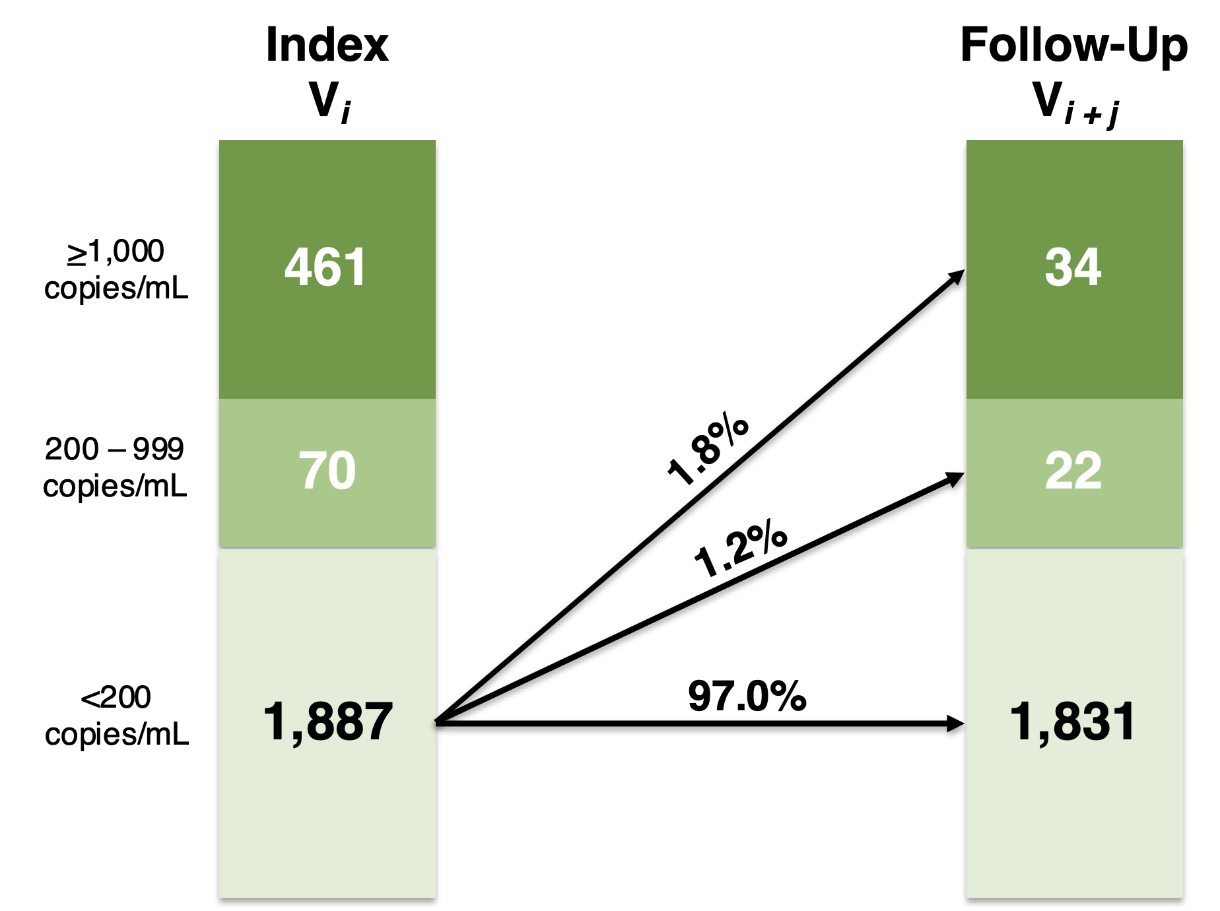

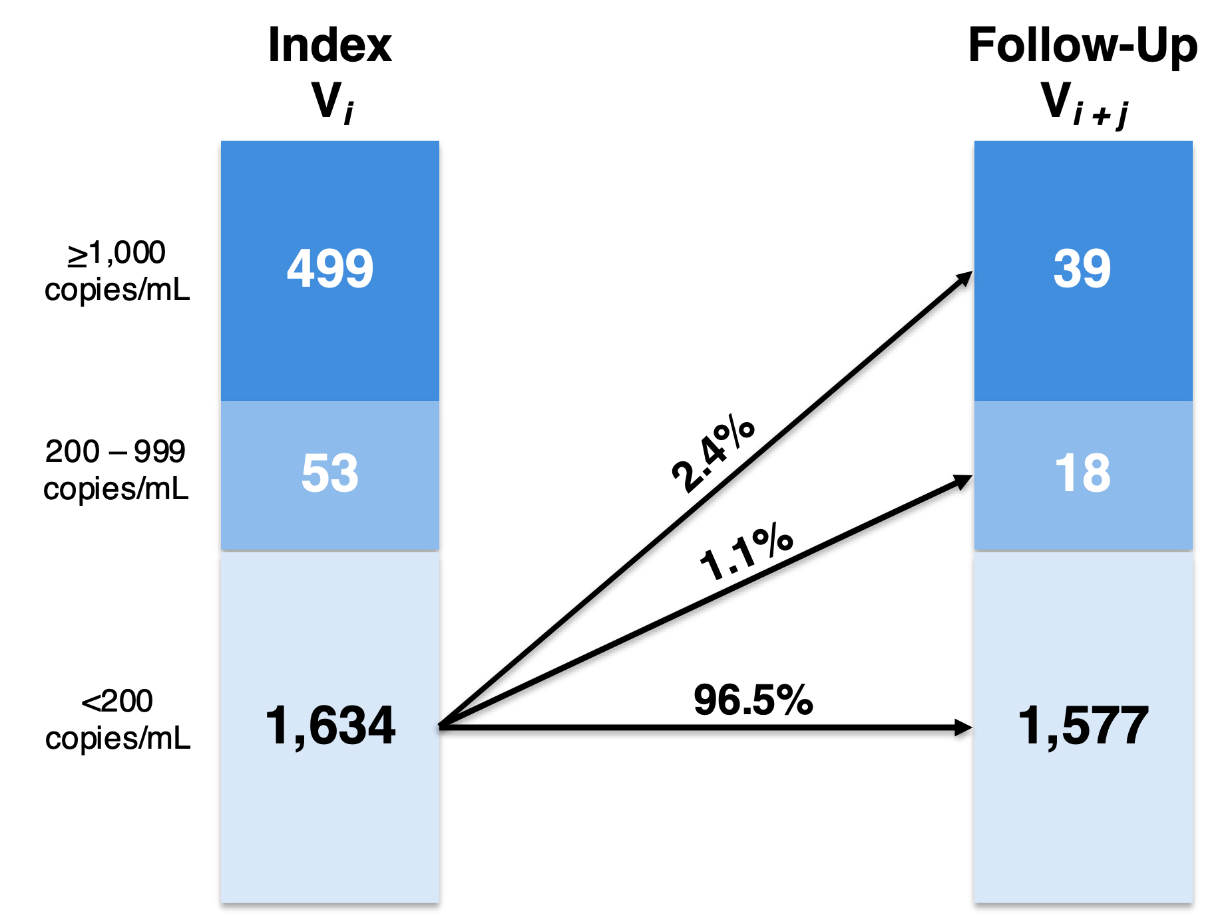


# **Table S2**. Visit-pair-level characteristics of participants exhibiting persistent high-level viraemia, by self-reported ART status.

| **Characteristics (*n*, %)** | **Total**  *N* = 429 | **ART <12 Months**  *n* = 340 (79.2%) | **ART >12 Months**  *n* = 89 (20.8%) | **χ²**  ***p*-value** |
| --- | --- | --- | --- | --- |
| ***Demographics*** |  |  |  |  |
| Index survey visit (calendar period) |  |  |  | **0.005** |
| Feb. 2015 – Sep. 2016 | 276 (64.3) | 230 (67.7) | 46 (51.7) |  |
| Oct. 2016 – May 2018 | 153 (35.7) | 110 (32.3) | 43 (48.3) |  |
| Age, in years (*median*, *IQR*)† | 31 (27–35) | 30 (26–35) | 33 (28–36) | **0.033** |
| Age group |  |  |  | 0.080 |
| 15-29 years | 190 (44.3) | 159 (46.8) | 31 (34.8) |  |
| 30-39 years | 174 (40.6) | 129 (37.9) | 45 (50.6) |  |
| 40-49 years | 65 (15.1) | 52 (15.3) | 13 (14.6) |  |
| Sex |  |  |  | **0.001** |
| Male | 245 (57.1) | 208 (61.2) | 37 (41.6) |  |
| Female | 184 (42.9) | 132 (38.8) | 52 (58.4) |  |
| Currently marital status |  |  |  | 0.505 |
| Never married | 53 (12.4) | 45 (13.2) | 8 (9.0) |  |
| Currently married | 243 (56.6) | 189 (55.6) | 54 (60.7) |  |
| Previously married | 133 (31.0) | 106 (31.2) | 27 (30.3) |  |
| Completed education |  |  |  | 0.310 |
| No formal education | 27 (6.3) | 20 (5.9) | 17 (7.9) |  |
| Primary | 304 (70.9) | 237 (69.7) | 67 (75.3) |  |
| Secondary | 91 (21.2) | 76 (22.3) | 15 (16.8) |  |
| Technical/University | 7 (1.6) | 7 (2.1) | *n/a* |  |
| Primary occupation |  |  |  | 0.106 |
| Agriculture or housework | 135 (31.5) | 108 (31.8) | 27 (30.3) |  |
| Trading or shopkeeping | 83 (19.3) | 66 (19.4) | 17 (19.1) |  |
| Bar work, waitressing, or sex work | 20 (4.7) | 11 (3.2) | 9 (10.1) |  |
| Fishing-related occupation | 107 (24.9) | 87 (25.6) | 20 (22.5) |  |
| Other | 84 (19.6) | 68 (20.0) | 16 (18.0) |  |
| Religion |  |  |  | 0.404 |
| Catholic/Christian | 379 (87.3) | 297 (87.3) | 82 (92.1) |  |
| Muslim | 48 (11.2) | 41 (12.1) | 7 (7.9) |  |
| Other/none | 2 (0.5) | 2 (0.6) | *n/a* |  |
| Household wealth (quartile) |  |  |  | 0.360 |
| Lowest | 202 (47.1) | 156 (45.9) | 146 (51.7) |  |
| Low-middle | 85 (19.8) | 74 (21.8) | 11 (12.4) |  |
| High-middle | 98 (22.8) | 75 (22.0) | 23 (25.8) |  |
| Highest | 43 (10.0) | 34 (10.0) | 9 (10.1) |  |
| *Missing* | *1 (0.2)* | *1 (0.3)* | *n/a* |  |
| Migration |  |  |  | 0.506 |
| Long-term resident | 341 (79.5) | 268 (78.8) | 73 (82.0) |  |
| In-migrant | 88 (20.5) | 72 (21.2) | 16 (18.0) |  |
| Community type |  |  |  | 0.142 |
| Agrarian | 129 (30.1) | 103 (30.3) | 26 (29.2) |  |
| Trading | 87 (20.3) | 75 (22.1) | 12 (13.5) |  |
| Fishing | 213 (49.6) | 162 (47.6) | 51 (57.3) |  |
| ***Behavioral*** |  |  |  |  |
| Number of sexual partners |  |  |  | 0.496 |
| 0–1 | 276 (64.3) | 216 (63.5) | 60 (67.4) |  |
| >2 | 153 (35.7) | 124 (36.5) | 29 (32.6) |  |
| Condom use |  |  |  | 0.267 |
| No partners or permanent partners only | 273 (63.6) | 211 (62.1) | 62 (69.7) |  |
| Consistent use with casual partners | 73 (17.0) | 58 (17.0) | 15 (16.8) |  |
| Inconsistent use with casual partners | 83 (19.4) | 71 (20.9) | 12 (13.5) |  |
| Transactional sex |  |  |  | 0.193 |
| No | 243 (56.6) | 198 (58.2) | 45 (50.6) |  |
| Yes | 186 (43.4) | 142 (41.8) | 44 (49.4) |  |
| Any alcohol use consequences |  |  |  | **0.021** |
| No | 321 (74.8) | 246 (72.3) | 75 (84.3) |  |
| Yes | 108 (25.2) | 94 (27.6) | 15 (15.7) |  |
| Illicit drug use |  |  |  | 0.487 |
| No | 403 (93.9) | 318 (93.5) | 85 (95.5) |  |
| Yes | 26 (6.1) | 22 (6.5) | 4 (4.5) |  |
| Intimate partner violence |  |  |  | 0.885 |
| No | 301 (70.2) | 238 (70.0) | 63 (70.8) |  |
| Yes | 128 (29.8) | 102 (30.0) | 26 (29.2) |  |

*Notes*: Individuals on ART <1 year also included those reporting never or previous ART use throughout the observation period. † *p*-values calculated using Wilcoxon rank-sum tests comparing median values with interquartile ranges (IQR). *^§^* Behavioral factors measured in the past year.

# **Table S3**. Weighted region-level prevalence of persistent high-level viraemia, by sex and calendar period.

| **Geo-masked Region** | **Survey Interval 1:**  Jun. 2015 to Sep. 2016 | | |  | **Survey Interval 2:**  Oct. 2016 to May 2018 | | |  | **Mann-Whitney U Test**  % Diff. (*p*-value)* | | |
| --- | --- | --- | --- | --- | --- | --- | --- | --- | --- | --- | --- |
|  | Males  *n* = 930 | Females  *n* = 1,558 | Total  *N* = 2,488 |  | Males  *n* = 779 | Females  *n* = 1,337 | Total  *N* = 2,116 |  | Males | Females | Total |
| Region A | 21.5  (16.4–26.7) | 7.7  (4.9–10.5) | 13.3  (10.6–16.0) |  | 12.2  (7.7–16.6) | 7.5  (4.6–10.4) | 9.3  (6.8–11.8) |  | **–9.3**  **(0.010)** | –0.2  (0.567) | **–4.0**  **(0.015)** |
| Region B | 38.0  (22.9–53.0) | 12.7  (6.0–19.4) | 20.3  (13.5–27.0) |  | 13.6  (2.0–25.1) | 9.7  (3.2–16.3) | 10.9  (5.1–16.6) |  | **–24.4**  **(0.048)** | –3.0  (0.604) | –9.4  (0.094) |
| Region C | 5.8  (<0.1–14.0) | 11.0  (4.8–17.3) | 9.7  (4.6–14.8) |  | 15.0  (1.3–28.8) | 3.2  (<0.1–7.3) | 6.2  (1.4–11.0) |  | 9.2  (0.485) | –7.8  (0.094) | –3.5  (0.324) |
| Region D | 10.3  (3.1–17.5) | 4.7  (0.8–8.5) | 6.8  (3.2–10.4) |  | 11.8  (3.4–20.3) | 5.6  (1.2–10.0) | 7.7  (3.7–11.9) |  | 1.5  (0.949) | 0.9  (0.839) | 0.9  (0.891) |
| Region E | 17.2  (8.5–26.0) | 5.3  (2.1–8.5) | 8.5  (5.2–11.9) |  | 16.6  (8.1–25.1) | 4.4  (14.8–7.2) | 7.8  (4.6–11.0) |  | –0.6  (0.767) | –0.9  (0.444) | –0.7  (0.480) |
| Region F | 16.6  (12.5–20.6) | 8.2  (5.5–11.0) | 12.0  (9.6–14.4) |  | 8.6  (5.4–11.8) | 5.9  (3.4–8.5) | 7.2  (5.1–9.2) |  | **–8.0**  **(0.004)** | –2.3  (0.176) | **–4.8**  **(0.002)** |
| Region G | 11.3  (<0.1–22.8) | 1.9  (<0.1–5.6) | 5.2  (0.5–10.0) |  | 10.0  (<0.1–29.6) | 2.2  (<0.1–6.5) | 3.5  (<0.1–8.4) |  | –1.3  (1.000) | –0.3  (0.899) | –1.7  (0.745) |
| Region H | 15.0  (4.1–26.0) | 9.9  (4.8–15.2) | 11.2  (6.5–16.0) |  | 29.0  (9.6–48.4) | 6.5  (1.1–12.0) | 11.6  (5.3–17.8) |  | 14.0  (0.399) | –3.4  (0.251) | 0.4  (0.638) |
| Region I | 18.7  (8.7–28.6) | 10.3  (4.8–15.9) | 13.2  (8.2–18.2) |  | 11.9  (2.8–20.9) | 5.2  (0.8–9.6) | 7.5  (3.3–11.7) |  | –6.8  (0.219) | 1.6  (0.119) | **–5.7**  **(0.048)** |

***Bolded** values represent statistically significant region-level median differences in persistent high-level viraemia over time at the *p* < 0.05 level or below.

# **Table S4**. Weighted community-level prevalence of persistent high-level viraemia, by calendar period.

| **Geo-masked**  **Community** |  | **Survey Interval 1:**  Jun. 2015 to Sep. 2016 | | | | |  | **Survey Interval 2:**  Oct. 2016 to May 2018 | | | | |  | **Mann-Whitney**  **U Test** | |
| --- | --- | --- | --- | --- | --- | --- | --- | --- | --- | --- | --- | --- | --- | --- | --- |
|  |  | *N* | Weighted % | 95% CI | | |  | *N* | Weighted % | 95% CI | | |  | % Diff. | *p*-value* |
| 1 |  | 13 | 8.1 | <0.1 | to | 23.6 |  | *n/a* | *n/a* | *n/a* | | |  | *n/a* | *n/a* |
| 2 |  | 52 | 10.1 | 1.8 | to | 18.4 |  | 40 | 6.9 | <0.1 | to | 14.9 |  | –3.2 | 0.606 |
| 4 |  | 45 | 12.8 | 2.9 | to | 22.7 |  | 28 | 8.2 | <0.1 | to | 18.6 |  | –4.6 | 0.578 |
| 5 |  | 43 | 8.1 | <0.1 | to | 16.4 |  | 35 | 4.2 | <0.1 | to | 11.0 |  | –3.9 | 0.415 |
| 6 |  | 45 | 6.9 | <0.1 | to | 14.4 |  | 37 | 15.8 | 3.9 | to | 27.8 |  | 8.9 | 0.507 |
| 7 |  | 47 | 14.2 | 4.1 | to | 24.3 |  | 37 | 9.9 | 0.2 | to | 19.7 |  | –4.3 | 0.785 |
| 8 |  | 30 | 21.9 | 6.8 | to | 37.0 |  | 28 | 9.2 | <0.1 | to | 20.2 |  | –12.7 | 0.208 |
| 16 |  | 19 | 14.5 | <0.1 | to | 30.8 |  | 16 | 10.6 | <0.1 | to | 26.2 |  | –3.9 | 0.785 |
| 19 |  | 54 | 14.8 | 5.2 | to | 24.4 |  | 42 | 9.7 | 0.7 | to | 18.8 |  | –5.1 | 0.244 |
| 23 |  | 56 | 10.5 | 2.4 | to | 18.6 |  | 56 | 4.8 | <0.1 | to | 10.5 |  | –5.7 | 0.299 |
| 24 |  | 73 | 9.6 | 2.8 | to | 16.4 |  | 47 | 6.8 | <0.1 | to | 14.2 |  | –2.8 | 0.537 |
| 29 |  | 31 | 3.3 | <0.1 | to | 9.6 |  | 28 | 7.3 | <0.1 | to | 17.1 |  | 4.0 | 0.498 |
| 33 |  | 29 | 8.4 | <0.1 | to | 18.7 |  | 21 | 0 | *n/a* | | |  | –8.4 | 0.224 |
| 34 |  | 73 | 6.6 | 0.9 | to | 12.3 |  | 65 | 10.8 | 3.2 | to | 18.4 |  | 4.2 | 0.608 |
| 36 |  | 31 | 3.3 | <0.1 | to | 9.7 |  | 22 | 0 | *n/a* | | |  | –3.3 | 0.400 |
| 38 |  | 609 | 11.9 | 9.4 | to | 14.5 |  | 549 | 7.3 | 5.2 | to | 9.5 |  | **–4.6** | **0.007** |
| 40 |  | 50 | 7.7 | 0.2 | to | 15.2 |  | 51 | 10.2 | 1.8 | to | 18.6 |  | 2.5 | 0.752 |
| 55 |  | 38 | 8.9 | <0.1 | to | 18.1 |  | 20 | 10.0 | <0.1 | to | 23.5 |  | 1.1 | 0.788 |
| 57 |  | 24 | 9.4 | <0.1 | to | 21.4 |  | 21 | 19.0 | 1.8 | to | 36.2 |  | 9.6 | 0.531 |
| 58 |  | 13 | 17.9 | <0.1 | to | 39.7 |  | 15 | 14.1 | <0.1 | to | 32.3 |  | –3.8 | 0.879 |
| 62 |  | 36 | 3.0 | <0.1 | to | 8.6 |  | 31 | 0 | *n/a* | | |  | –3.0 | 0.353 |
| 74 |  | 10 | 20.2 | <0.1 | to | 46.5 |  | 11 | 24.5 | <0.1 | to | 51.1 |  | 4.3 | 0.918 |
| 77 |  | 23 | 17.8 | 1.8 | to | 33.8 |  | 26 | 7.3 | <0.1 | to | 17.4 |  | –10.5 | 0.306 |
| 89 |  | 28 | 6.6 | <0.1 | to | 16.0 |  | 26 | 9.2 | <0.1 | to | 20.5 |  | 2.6 | 0.939 |
| 106 |  | 60 | 24.4 | 13.4 | to | 35.4 |  | 50 | 13.0 | 3.6 | to | 22.4 |  | –11.4 | 0.184 |
| 107 |  | 31 | 7.4 | <0.1 | to | 16.8 |  | 22 | 7.3 | <0.1 | to | 18.4 |  | –0.1 | 0.770 |
| 108 |  | 46 | 6.6 | <0.1 | to | 13.9 |  | 57 | 7.4 | 0.5 | to | 14.3 |  | 0.8 | 0.787 |
| 120 |  | 9 | 0 | *n/a* | | |  | 8 | 18.7 | <0.1 | to | 47.6 |  | 18.7 | 0.289 |
| 370 |  | 43 | 15.4 | 4.5 | to | 26.4 |  | 27 | 8.1 | <0.1 | to | 18.5 |  | –7.3 | 0.406 |
| 391 |  | 27 | 22.8 | 6.7 | to | 39.0 |  | 25 | 0 | *n/a* | | |  | **–22.8** | **0.013** |
| 602 |  | 99 | 13.2 | 6.5 | to | 19.9 |  | 88 | 3.4 | <0.1 | to | 7.2 |  | **–9.8** | **0.031** |
| 754 |  | 42 | 10.4 | 1.1 | to | 19.8 |  | 16 | 21.3 | 0.5 | to | 42.0 |  | 10.9 | 0.742 |
| 770 |  | 127 | 11.5 | 5.9 | to | 17.1 |  | 132 | 9.8 | 4.7 | to | 14.9 |  | –1.7 | 0.606 |
| 771 |  | 302 | 11.8 | 8.1 | to | 15.4 |  | 246 | 6.1 | 3.1 | to | 9.1 |  | **–5.7** | **0.027** |
| 772 |  | 26 | 15.5 | 1.3 | to | 29.6 |  | 20 | 4.1 | <0.1 | to | 13.1 |  | –11.4 | 0.267 |
| 773 |  | 31 | 23.0 | 7.9 | to | 38.1 |  | 27 | 28.9 | 11.5 | to | 46.4 |  | 5.9 | 0.790 |
| 774 |  | 117 | 15.9 | 9.2 | to | 22.6 |  | 102 | 11.0 | 4.9 | to | 17.1 |  | –4.9 | 0.194 |

***Bolded** values represent statistically significant community-level median differences in persistent high-level viraemia over time at the *p* < 0.05 level or below.

# **Table S5**. Risk of persistent high-level HIV viraemia (>1,000 copies/mL) relative to sustained or new/renewed low-level viraemia or suppression, by sex.

|  | **Males (*N* = 1,146)** | |  | **Females (*N* = 1,882)** | |
| --- | --- | --- | --- | --- | --- |
| **Characteristics** | RR (95% CI) | adjRR (95%CI) |  | RR (95% CI) | adjRR (95% CI) |
| ***Demographics*** |  |  |  |  |  |
| Age group |  |  |  |  |  |
| 15-29 years | **3.07 (2.17–4.35)** | **2.45 (1.71–3.52)** |  | **4.82 (2.87–8.08)** | **3.59 (2.10–6.14)** |
| 30-39 years | **1.49 (1.07–2.07)** | **1.41 (1.02–1.96)** |  | **2.34 (1.42–3.85)** | **2.04 (1.23–3.37)** |
| 40-49 years | *Ref*. | *Ref*. |  | *Ref*. | *Ref*. |
| Currently marital status |  |  |  |  |  |
| Never married | **2.14 (1.50–3.06)** | 1.35 (0.92–1.98) |  | **1.59 (1.02–2.51)** | 1.33 (0.87–2.04) |
| Currently married | *Ref*. | *Ref*. |  | *Ref*. | *Ref*. |
| Previously married | 1.26 (0.94–1.68) | 1.21 (0.92–1.60) |  | 0.83 (0.59–1.17) | 0.88 (0.64–1.23) |
| Completed education |  |  |  |  |  |
| No formal education | 0.77 (0.47–1.27) |  |  | 0.99 (0.53–1.85) | 1.17 (0.63 – 2.18) |
| Primary | *Ref*. |  |  | *Ref*. | *Ref*. |
| Secondary | 0.99 (0.65–1.49) |  |  | **2.21 (1.52–3.22)** | **1.92 (1.34–2.74)** |
| Technical/University | 1.07 (0.42–2.68) |  |  | 0.51 (0.13–2.05) | 0.64 (0.16–2.58) |
| Migration |  |  |  |  |  |
| Long-term resident | *Ref*. | *Ref*. |  | *Ref*. | *Ref*. |
| In-migrant | **1.64 (1.25–2.16)** | 1.21 (0.92–1.58) |  | **1.52 (1.17–1.97)** | 1.11 (0.86–1.43) |
| ***Behavioral*** |  |  |  |  |  |
| Condom use |  |  |  |  |  |
| No partners or permanent partners only | *Ref*. | *Ref*. |  | *Ref*. | *Ref*. |
| Consistent use with casual partners | 1.25 (0.93–1.69) | 1.12 (0.84–1.50) |  | **1.57 (1.52–2.19)** | 1.31 (0.91–1.89) |
| Inconsistent use with casual partners | **1.72 (1.31–2.25)** | **1.38 (1.05–1.80)** |  | 1.30 (0.94–1.80) | 1.15 (0.81–1.63) |
| Transactional sex |  |  |  |  |  |
| No | *Ref*. |  |  | *Ref*. | *Ref*. |
| Yes | 1.00 (0.77–1.30) |  |  | **1.35 (1.04–1.74)** | 1.15 (0.87–1.53) |
| Hazardous alcohol use | **1.11 (1.03–1.19)** | **1.08 (1.01–1.16)** |  | 1.15 (0.99–1.32) | 1.08 (0.95–1.23) |
| Intimate partner violence |  |  |  |  |  |
| None | *Ref*. |  |  | *Ref*. | *Ref*. |
| Any | 1.17 (0.94–1.46) |  |  | **1.36 (1.05–1.75)** | 1.09 (0.84–1.41) |

*Notes*: Risk ratios (RR) and 95% confidence intervals (95%CI) were estimated using Poisson regression with generalized estimating equations, exchangeable covariance matrices, and robust standard errors. Multivariable models were adjusted for survey interval (calendar period) of index visit and all covariates displayed in the columns presenting adjusted results. Sustained or new/renewed low-level viraemia or suppression was defined as <1,000 copies/mL across visits or at follow-up only. **Bolded** values represent risk ratios of persistent HIV viraemia that were significantly different from the null value of 1 at the *p* < 0.05 level or below. Behavioral factors measured in the past year.

# **Table S6**. Risk of persistent high-level HIV viraemia (>1,000 copies/mL) relative to sustained or new/renewed low-level viraemia or suppression, by community type.

|  | **Inland Communities (*N* = 1,560)** | |  | **Fishing Communities (*N* = 1,468)** | |
| --- | --- | --- | --- | --- | --- |
| **Characteristics** | RR (95% CI) | adjRR (95% CI) |  | RR (95% CI) | adjRR (95% CI) |
| ***Demographics*** |  |  |  |  |  |
| Age group |  |  |  |  |  |
| 15-29 years | **3.31 (2.30–4.76)** | **3.42 (2.38–4.93)** |  | **3.18 (2.00–5.07)** | **3.00 (1.86–4.86)** |
| 30-39 years | 1.32 (0.92–1.89) | 1.33 (0.92–1.90) |  | **2.23 (1.44–3.44)** | **2.26 (1.46–3.49)** |
| 40-49 years | *Ref*. | *Ref*. |  | *Ref*. | *Ref*. |
| Sex |  |  |  |  |  |
| Male | **1.97 (1.46–2.66)** | **2.16 (1.59–2.94)** |  | **2.19 (1.58–3.04)** | **2.67 (1.94–3.67)** |
| Female | *Ref.* | *Ref.* |  | *Ref.* | *Ref.* |
| Currently marital status |  |  |  |  |  |
| Never married | 1.44 (0.99–2.11) |  |  | **2.43 (1.53–3.86)** | 1.55 (0.96–2.52) |
| Currently married | *Ref*. |  |  | *Ref*. | *Ref*. |
| Previously married | 0.77 (0.55–1.08) |  |  | 1.20 (0.88–1.64) | 1.21 (0.91–1.62) |
| Completed education |  |  |  |  |  |
| No formal education | 1.06 (0.55–2.05) |  |  | 0.74 (0.45–1.21) | 0.98 (0.60–1.58) |
| Primary | *Ref*. |  |  | *Ref*. | *Ref*. |
| Secondary | 1.37 (0.96–1.96) |  |  | **1.61 (1.05–2.47)** | **1.69 (1.14–2.51)** |
| Technical/University | 0.57 (0.21–1.52) |  |  | 1.87 (0.53–6.59) | 3.10 (0.89–10.79) |
| Household wealth |  |  |  |  |  |
| Lowest | **1.45 (1.00–2.14)** | **1.59 (1.09–2.33)** |  | 0.80 (0.30–1.65) |  |
| Low-middle | **1.60 (1.12–2.29)** | 1.38 (0.97–1.97) |  | 0.52 (0.24–1.17) |  |
| High-middle | **1.52 (1.08–2.12)** | **1.38 (1.01–1.88)** |  | 0.67 (0.29–1.54) |  |
| Highest | *Ref*. | *Ref*. |  | *Ref*. |  |
| Migration |  |  |  |  |  |
| Long-term resident | *Ref*. | *Ref*. |  | *Ref*. | *Ref*. |
| In-migrant | **1.55 (1.17–2.05)** | 1.17 (0.90–1.52) |  | **1.48 (1.13–1.93)** | 1.17 (0.88–1.54) |
| ***Behavioral*** |  |  |  |  |  |
| Number of past-year sexual partners |  |  |  |  |  |
| 0–1 | *Ref*. | *Ref*. |  | *Ref*. | *Ref*. |
| >2 | **1.44 (1.10–1.90)** | 0.91 (0.66–1.24) |  | **1.42 (1.07–1.87)** | 0.80 (0.59–1.09) |
| Condom use |  |  |  |  |  |
| No partners or permanent partners only | *Ref*. | *Ref*. |  | *Ref*. | *Ref*. |
| Consistent use with casual partners | **1.60 (1.13–2.27)** | 1.24 (0.87–1.77) |  | **1.44 (1.04–2.00)** | 1.33 (0.92–1.91) |
| Inconsistent use with casual partners | **1.38 (1.01–1.88)** | 1.29 (0.93–1.78) |  | **1.87 (1.38–2.54)** | 1.44 (1.02–2.02) |
| Hazardous alcohol use | **1.25 (1.13–1.37)** | **1.17 (1.08–1.27)** |  | **1.11 (1.02–1.21)** | 1.03 (0.95–1.12) |
| Intimate partner violence |  |  |  |  |  |
| None | *Ref*. |  |  | *Ref*. | *Ref*. |
| Any | 1.15 (0.89–1.48) |  |  | **1.32 (1.05–1.66)** | 1.16 (0.92–1.47) |

*Notes*: Inland communities include all mainland non-fishing (i.e., agrarian and trading) communities. Risk ratios (RR) and 95% confidence intervals (95%CI)

were estimated using Poisson regression with generalized estimating equations, exchangeable covariance matrices, and robust standard errors. Multivariable

models were adjusted for survey interval (calendar period) of index visit and all covariates displayed in the columns presenting adjusted results. Sustained or new/renewed low-level viraemia or suppression was defined as <1,000 copies/mL across visits or at follow-up only. **Bolded** values represent risk ratios of persistent HIV viraemia that were significantly different from the null value of 1 at the *p* < 0.05 level or below. Behavioral factors measured in the past year.

# **Table S7**. Descriptive sample statistics at the index visit for participants contributing one visit-pair (two visits) versus two visit-pairs (three visits) to the analysis—2015 to 2020.

| **Characteristics (*n*, %)** | **1 Visit-Pair**  *n* = 1,556 (50.5%) | **2 Visit-Pairs**  *n* = 1,524 (49.5%) | **Total**  *N* = 3,080 | **χ²**  ***p*-value***** |
| --- | --- | --- | --- | --- |
| ***Demographics*** |  |  |  |  |
| Age, in years (*median*, *IQR*)† | 33 (28–39) | 35 (30–40) | 34 (28–39) | **<0.001** |
| Age group |  |  |  | **<0.001** |
| 15-29 years | 528 (33.9) | 380 (24.9) | 908 (29.5) |  |
| 30-39 years | 661 (42.5) | 760 (49.9) | 1,421 (46.1) |  |
| 40-49 years | 367 (23.6) | 384 (25.2) | 751 (24.4) |  |
| Sex |  |  |  | **0.001** |
| Male | 637 (40.9) | 536 (35.2) | 1,173 (38.1) |  |
| Female | 919 (59.1) | 988 (64.8) | 1,907 (61.9) |  |
| Currently marital status |  |  |  | **0.007** |
| Never married | 133 (8.5) | 88 (5.8) | 221 (7.2) |  |
| Currently married | 894 (57.5) | 929 (61.0) | 1,823 (60.0) |  |
| Previously married | 529 (34.0) | 507 (33.3) | 1,036 (33.6) |  |
| Educational attainment |  |  |  | 0.730 |
| No formal education | 132 (8.5) | 128 (8.4) | 260 (8.5) |  |
| Primary | 1,140 (73.2) | 1,112 (73.0) | 2,252 (73.1) |  |
| Secondary | 241 (15.5) | 250 (16.4) | 491 (15.9) |  |
| Technical/University | 43 (2.8) | 23 (2.2) | 77 (2.5) |  |
| Primary occupation |  |  |  | **0.004** |
| Agriculture or housework | 511 (32.8) | 590 (38.7) | 1,101 (35.8) |  |
| Trading or shopkeeping | 323 (20.8) | 314 (20.6) | 637 (20.7) |  |
| Bar work, waitressing, or sex work | 136 (8.7) | 125 (8.2) | 261 (8.5) |  |
| Fishing-related occupation | 292 (18.8) | 227 (14.9) | 519 (16.8) |  |
| Other | 294 (18.9) | 268 (17.6) | 562 (18.2) |  |
| Religion |  |  |  | 0.260 |
| Catholic/Christian | 1,356 (87.2) | 1,348 (88.4) | 2,704 (87.8) |  |
| Muslim | 192 (12.3) | 164 (10.8) | 356 (11.6) |  |
| Other/none | 8 (0.5) | 12 (0.8) | 20 (0.6) |  |
| Household wealth (quartile) |  |  |  | **0.048** |
| Lowest | 685 (44.0) | 646 (42.4) | 1,331 (43.2) |  |
| Low-middle | 308 (19.8) | 346 (22.7) | 654 (21.2) |  |
| High-middle | 343 (22.0) | 327 (21.5) | 670 (21.8) |  |
| Highest | 214 (13.8) | 205 (13.4) | 419 (13.6) |  |
| *Missing* | *6 (0.4)* | *n/a* | *6 (0.2)* |  |
| Migration |  |  |  | **<0.001** |
| Long-term resident | 1,119 (71.9) | 1,335 (87.6) | 2,454 (79.7) |  |
| In-migrant | 437 (28.1) | 189 (12.4) | 626 (20.3) |  |
| Community type |  |  |  | **<0.001** |
| Agrarian | 411 (26.4) | 522 (24.2) | 933 (30.3) |  |
| Trading | 339 (21.8) | 312 (20.5) | 651 (21.1) |  |
| Fishing | 806 (51.8) | 690 (45.3) | 1,496 (48.6) |  |
| ***Behavioral*** |  |  |  |  |
| Number of sexual partners |  |  |  | **0.001** |
| 0–1 | 1,099 (70.6) | 1,157 (75.9) | 2,256 (73.2) |  |
| >2 | 457 (29.4) | 367 (24.1) | 824 (26.8) |  |
| Condom use |  |  |  | 0.222 |
| No partners or permanent partners only | 1,130 (72.6) | 1,102 (72.3) | 2,232 (72.5) |  |
| Consistent use with casual partners | 216 (13.9) | 189 (12.4) | 405 (13.1) |  |
| Inconsistent use with casual partners | 210 (13.5) | 233 (15.3) | 443 (14.4) |  |
| Transactional sex |  |  |  | 0.178 |
| No | 830 (53.3) | 776 (50.9) | 1,606 (52.1) |  |
| Yes | 726 (46.7) | 748 (49.1) | 1,474 (47.9) |  |
| Any alcohol use consequences |  |  |  | 0.182 |
| No | 1,449 (93.1) | 1,437 (94.3) | 2,886 (93.7) |  |
| Yes | 107 (6.9) | 87 (5.7) | 194 (6.3) |  |
| Illicit drug use |  |  |  | **<0.001** |
| No | 1,464 (94.1) | 1,481 (97.2) | 2,945 (95.6) |  |
| Yes | 92 (5.9) | 43 (2.8) | 135 (4.4) |  |
| ***HIV-related*** |  |  |  |  |
| ART use history (self-reported) |  |  |  | **<0.001** |
| Never | 530 (34.1) | 401 (26.3) | 931 (30.2) |  |
| Currently or previously | 1,026 (65.9) | 1,123 (73.7) | 2,149 (69.8) |  |
| Visit-pair viral load |  |  |  | **<0.001** |
| Durable VLS | 1,006 (64.7) | 1,107 (72.6) | 2,113 (68.6) |  |
| New/renewed VLS | 262 (16.8) | 225 (14.8) | 487 (15.8) |  |
| Viral rebound | 44 (2.8) | 32 (2.1) | 76 (2.5) |  |
| Persistent viraemia | 244 (15.7) | 160 (10.5) | 404 (13.1) |  |

**p*-values calculated using Pearson’s chi-square test of association, unless otherwise specified. † *p*-values calculated using Wilcoxon rank-sum tests comparing median values and interquartile ranges (IQR). *^§^* Behavioral factors measured in the past year. *Notes*: VLS was defined using an HIV RNA cutpoint of <200 copies/mL. Demographic variables were derived from the index visit (V*_i_*) in the visit-pair, and behavioral factors were derived from the follow-up visit (V*_i+j_*) in the visit-pair.

# **Figure S3.** Box plots of stabilized inverse probability of selection and censoring weights, by number of visit-pairs contributed to the analysis.

**
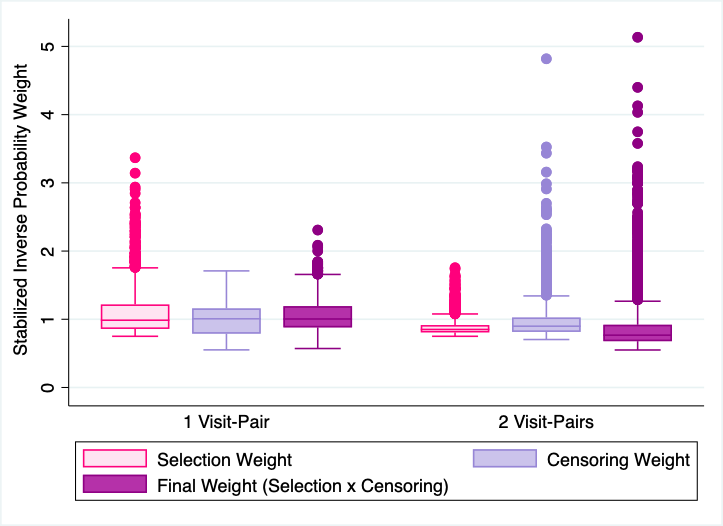
**
